# McEnhancer: predicting gene expression via semi-supervised assignment of enhancers to target genes

**DOI:** 10.1186/s13059-017-1316-x

**Published:** 2017-10-26

**Authors:** Dina Hafez, Aslihan Karabacak, Sabrina Krueger, Yih-Chii Hwang, Li-San Wang, Robert P. Zinzen, Uwe Ohler

**Affiliations:** 1 Department of Computer Science, Duke University, Durham, 27708 NC USA; 2 Berlin Institute for Medical Systems Biology, Max Delbrück Center for Molecular Medicine, Berlin, 13125 Germany; 3 Departments of Biology and Computer Science, Humboldt University, Berlin, 10099 Germany; 4 Genomics and Computational Biology Graduate Program, University of Pennsylvania, Philadelphia, 19104 PA USA

**Keywords:** Interpolated Markov model, Enhancer to target gene assignment, Gene expression, *Drosophila melanogaster*, Gene regulation, Machine learning, Semi-supervised model

## Abstract

Transcriptional enhancers regulate spatio-temporal gene expression. While genomic assays can identify putative enhancers en masse, assigning target genes is a complex challenge. We devised a machine learning approach, McEnhancer, which links target genes to putative enhancers via a semi-supervised learning algorithm that predicts gene expression patterns based on enriched sequence features. Predicted expression patterns were 73–98% accurate, predicted assignments showed strong Hi-C interaction enrichment, enhancer-associated histone modifications were evident, and known functional motifs were recovered. Our model provides a general framework to link globally identified enhancers to targets and contributes to deciphering the regulatory genome.

**Electronic supplementary material:**

The online version of this article (doi:10.1186/s13059-017-1316-x) contains supplementary material, which is available to authorized users.

## Background

In complex metazoan genomes, the space dedicated to encoding gene expression typically exceeds the space to encode the actual genes. Identifying and interpreting these “non-coding” regulatory regions is a crucial step towards understanding gene regulation. Transcriptional regulatory regions encompass gene-proximal promoter regions as well as gene-distal regions such as *enhancers* and *insulators*, which can regulate target genes across long distances [1–3].

For many years, enhancers were primarily identified by functional dissection and reporter assays. Recent methods to identify enhancer candidates on a genomic scale are mostly based on chromatin features. Assays such as DNase-seq or ATAC-seq have been used to map open chromatin regions across hundreds of human cell lines or diverse model systems [4–6]. Complemented by histone modification mapping, such methods were used as a strong indicator for active enhancers; see, for example, [7–9].

Enhancer sequences contain short DNA motifs that work as binding sites for sequence-specific transcription factors (TFs), and the combination of recruited (co-) activators and repressors determines the activity of the enhancer [10]. Under the assumption that genes with correlated expression patterns tend to be regulated by similar TFs, co-expressed genes are more likely to be controlled by enhancers that contain a similar set of transcription factor binding sites (TFBSs) [3]. Regulatory regions are referred to as *cis*-regulatory modules (CRMs); they involve the dynamic interplay between several TFs as well as nucleosome and chromatin organizing proteins within a defined genomic interval. In human, various TFBSs were shown to be conserved among co-expressed genes in promoter regions [11]. Moreover, enhancers that direct similar expression share common TFBSs. The use of regulatory sequence features in putative enhancers showed promising results in predicting cell-type-specific gene expression [8, 12]. In previous work, we assigned human cell-type-specific DNaseI hypersensitive (DHS) regions to their closest gene, and scores for TFBSs in the associated DHSs were used as features in sparse logistic regression classifiers to discriminate between different expression patterns [13]. The additional information gained by incorporating features from distal enhancers significantly exceeded the use of proximal promoters only.

**Fig. 1:**
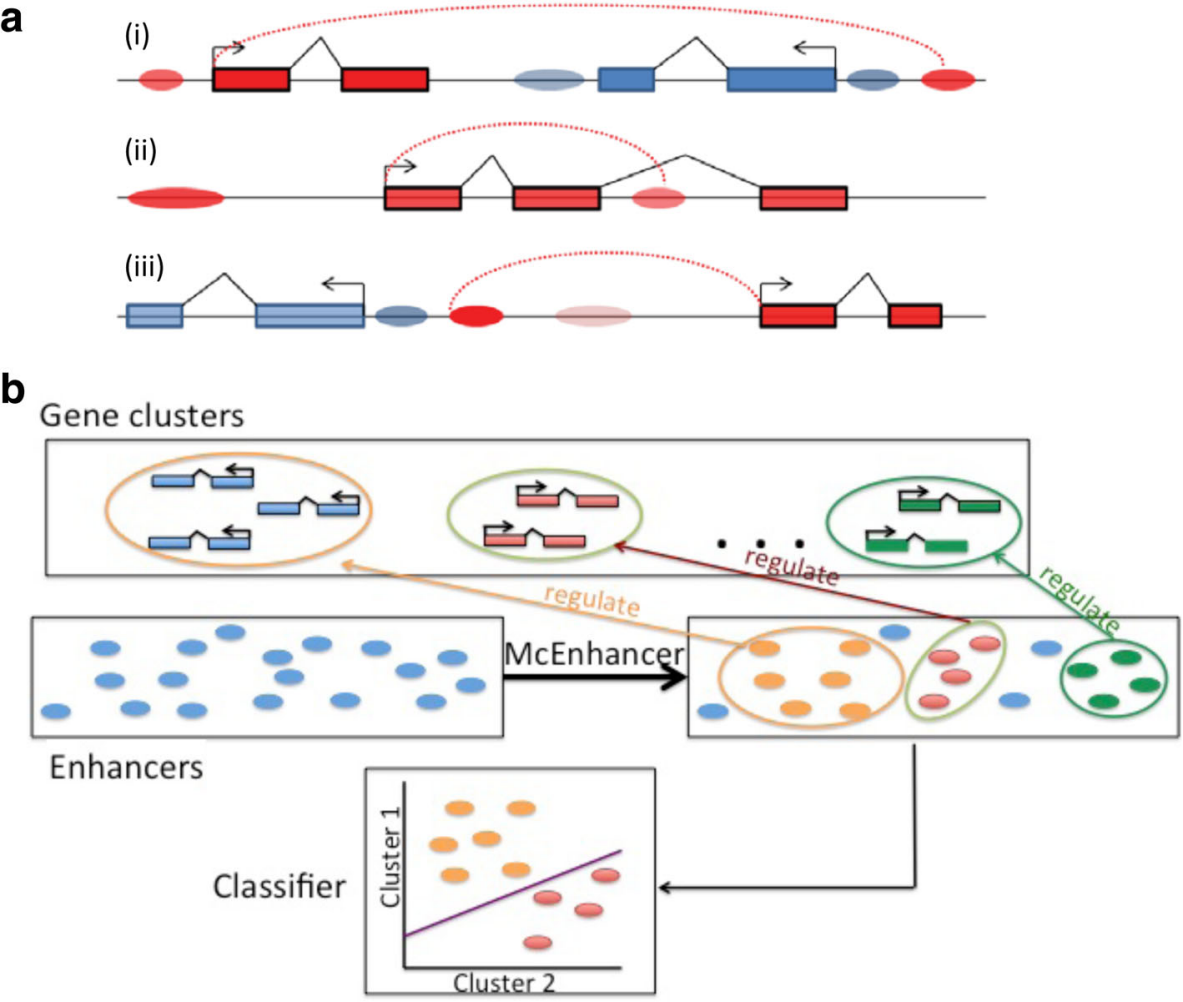
Possible enhancer-gene interaction dynamics and problem definition. **a** Current understanding of interaction dynamics between enhancers and their target genes: (i) An enhancer can regulate a faraway gene and not necessarily the closest one. Genes with different expression patterns are marked using different colors. Enhancers associated with a specific gene (or expression pattern) are marked with the same color of the target gene. (ii) An enhancer may exist in an intron of its host or a different target gene. (iii) A gene can be regulated by multiple enhancers. **b** Problem definition: genes with highly similar expression patterns are grouped into clusters. Enhancers are selected from the list of unlabeled sets and assigned a gene cluster label. As a final step, a logistic regression classifier is applied to test the ability of the sequence of selected enhancers to predict the genes’ expression patterns

While these results were encouraging, performance certainly suffered from the simplified and often incorrect assignment of enhancers to the closest gene. However, linking enhancers to their target genes is not straightforward. Figure 1 a illustrates our current understanding of expected enhancer-gene relations: Enhancers can be located in the vicinity of their target genes but do not necessarily regulate the closest gene; they can act across intervening genes to reach their targets; and they can exist in the introns of their target genes or even those of nearby genes. Furthermore, genes are often regulated by more than one enhancer. The *even-skipped* gene of *Drosophila* is one of the classic examples, where at least nine enhancers control expression in a spatially and temporally distinct fashion, but enhancers that independently direct largely similar expression patterns have also been described [3, 14, 15]. Finally, the relationship between enhancers and their target genes is not necessarily limited to one-to-one, as a single enhancer can target multiple genes [16].

To disentangle the complex relationships between regulatory regions and target genes, previous studies employed a number of different approaches. (1) Genome-wide techniques to capture long-range chromatin interactions, such as Hi-C [17] or ChIA-PET [18], may identify enhancer-gene relationships, based on the assumption that active enhancers direct target gene expression based on looping to the target promoter. However, the current scale of available mammalian datasets does not typically allow for detailed assignments at the needed kilobase resolution [19]. (2) Instead, several approaches assigned enhancers to target genes based on the correlation of gene expression and histone modifications in enhancers and putative target promoters. In one example, enhancer states were identified based on combinatorial patterns of chromatin marks using a multivariate hidden Markov model (HMM), and enriched TF motifs in these enhancers were matched against gene expression profiles [20]. This model was used to identify potential long-range interaction between pairs of predicted enhancers and genes across multiple cell lines in human [21]. PreSTIGE (Predicting Specific Tissue Interactions of Genes and Enhancers) is another approach that associates H3K4me1 ChIP-seq with RNA-seq expression levels for genes that are specifically expressed in different cell types [22]. However, all of these models depend on enhancer-associated chromatin marks, which require high-resolution data at multiple conditions (e.g., time points and cell types). (3) Alternatively, expression quantitative trait loci (eQTLs) have also been used to link enhancers to target genes, via correlating sequence variation to changes in gene expression levels [23]. An integrative random forest model to predict genes associated with human eQTLs was introduced by [24]. However, given the intrinsic challenges that usually come with eQTL analysis, such as the large number of tests needed to identify associations between gene expression and single-nucleotide polymorphism, such an approach has limited practicality for genome-wide implementation. (4) Finally, models on specific expression patterns, for which relevant TFs and genes are well known, have been utilized to link specific sets of enhancers to their target genes. For *Drosophila* [25], a Bayesian approach used the occupancy profile of different TFs, spatio-temporal activity for CRMs, and their genomic distances, along with occupancy peaks for six insulator binding proteins and H3K4me3 profiles at promoters. This model suggested that an enhancer could regulate multiple genes and that enhancers that are 50 kb away from a gene transcription start site (TSS) could still have influence on that gene. Recently, a linear model integrating TF motif score, expression domain, and chromatin accessibility data across developmental stages has been shown to predict enhancer activities [26]. Again, prior knowledge of TFs and their DNA binding motifs is required.

In this paper, we utilize a different premise to develop a predictive model linking enhancers to target genes, assuming that similar spatio-temporal expression observed in target genes is causally connected to shared sequence features (e.g., TFBSs) present in their enhancers. We focus our efforts on fly embryonic enhancers: The fly genome is compact, such that enhancers are frequently close to multiple genes (e.g., within a few kilobases), and one can therefore not assume that the closest promoter is always the correct one [27, 28]. Furthermore, while intricate expression patterns have been extensively mapped, chromatin data is not available at matching spatio-temporal resolution that would allow for the application of chromatin-state-correlation approaches. Classifying gene expression patterns based on our assigned enhancers dramatically improves upon baseline approaches. We demonstrate the success of our strategy by agreement with Hi-C data, interpret informative sequence features, and validate a subset of candidates with reporter constructs.

## Approach

### Problem definition

Previous approaches that assign enhancers to target genes largely rely on correlation between gene expression and chromatin state: They look for cases in which changes in enhancer histone modifications correlate with changes in transcription and/or promoter histone modifications, e.g., over a set of conditions (such as cell lines) for which both expression and chromatin states have been determined. While intricate spatio-temporal gene expression patterns have been measured in whole intact organisms such as fly embryos, plant roots, or mouse brains, they do not come with epigenetic information at the same matching resolution. This constitutes a bottleneck that limits our ability to interpret and decode functional regulatory regions.

However, existing approaches do not utilize a critical feature of gene regulation, namely that co-regulated genes can be assumed to be targeted by the same (subset of) regulatory factors, and that this is reflected in the co-occurrence of sequence features in the regions to which they bind [3]. We are therefore proposing to fill this gap via predictive sequence models. Starting from a set of genes with highly similar expression patterns, and a candidate list of enhancers in their genomic neighborhood, the task is to infer a yes/no label for each enhancer indicating its involvement in regulating the genes (Fig. 1 b). We can then demonstrate the validity of these assignments via the improved ability to predict the expression pattern from the sequences of the selected enhancers. This strategy can be thought of as higher-level motif finding. In the traditional motif finding problem, one is asked to determine the locations of a common motif that is shared in a set of regulatory regions. Here, there is similarly a hidden variable indicating whether an enhancer regulates a specific target gene, but in place of a motif model, we learn a model for the set of selected enhancers.

### Choice of biological model system and data

In this study, we specify a solution of this general idea for the model system of the fruit fly. *Drosophila melanogaster* has a compact genome, and an immense volume of data has been accumulated about fly biology generally and gene regulation specifically. *Drosophila* shares many similar regulatory features and pathways with humans on the molecular level, but its short life cycle and the available genetic and transgenic tools allow us to experimentally validate specific candidates. These aspects make *Drosophila* a powerful model organism for testing predictive models of gene expression regulation.

Specifically, we were able to use the following resources:

1. **Genome-wide spatio-temporal expression profiles of** ***Drosophila*** **embryonic development.** Over the past 15 years, gene expression patterns for thousands of genes have been curated by the Berkeley *Drosophila* Genome Project [29]. Images were taken at multiple time points during development and manually annotated with a controlled vocabulary describing the patterns [29]. A subset of these data (44% of the 13,659 protein-coding genes) has been used in conjunction with conventional time-course gene expression data to cluster genes into 39 patterns based on a combination of micro-array expression data as well as the vocabulary, ranging from 3 to 365 unique genes per cluster [30]. Out of these clusters, 29 clusters define restricted expression patterns; the remaining 10 broad clusters are grouped together and referred to as ubiquitously expressed genes.
2. **Genome-wide data on putative enhancers active during** ***Drosophila*** **embryogenesis.** A curated set of all possible enhancers during *Drosophila melanogaster* embryogenesis is not available, but active enhancers are known to be depleted of nucleosomes so as to make the DNA accessible and “open.” To identify accessible DNA regions in the genome, DNaseI digestion and high-throughput sequencing (DNase-seq) are usually used to mark the locations of open chromatin along the genome [4, 31, 32]. DHSs have proven to be well correlated with diverse classes of *cis*-regulatory regions, including promoters, enhancers, insulators, and other sites of regulatory factor occupancy [33–35]. It was also shown that active regulatory regions interacting with gene promoters are usually enriched for DHSs [36].
  By considering the joint set of high-resolution in vivo DNase-seq assays profiled at different stages of embryogenesis [37], DHSs could be used to map the locations of regulatory regions, decreasing the search space (to 6.4% of the genome) and increasing the probability of predicting regulatory regions that are functional. Note that in our approach, chromatin data is solely used to define a superset of putative enhancers; it does not represent condition-specific activity information to link enhancers to targets.
3. **Initialization of enhancer models.** While motif finding in general does not require prior knowledge to initialize models, available resources on gene regulation in *Drosophila* allow us to make use of information on validated enhancers and the expression patterns they confer. The most detailed, manually curated available resource for experimentally verified fly CRMs, along with their associated genes, is the Regulatory Element Database for *Drosophila* (REDfly) [38]. An additional, more recent set of known enhancer-gene pairs has been determined via high-throughput enhancer trapping [39]. In this Vienna Tiles (VT) library, each line contains a transcriptional reporter construct with an ∼2kb candidate enhancer. These tested fragments were selected to gain a largely unbiased picture of the frequency and distribution of regulatory activity in the non-coding, non-repetitive genome (around 13.5%). In situ images for each transgenic line were acquired, and the enhancer activity patterns were manually annotated using a controlled vocabulary. Enhancers were then assigned to genes by manually matching their activities with gene expression patterns. Overlapping these known CRMs from REDfly or the VT library with open chromatin regions allows us to assign a small subset of DHSs in the expression clusters to their target genes, enabling us to address the problem in a semi-supervised framework.

### The McEnhancer model

In principle, one can define a complete system for enhancer-gene assignment by including assignments as hidden variables in a probabilistic sequence-based model of gene expression. Sampling over putative assignments of enhancers to genes, for all expression patterns simultaneously, should allow for computing posterior probabilities of assignments leading to optimal classification.

**Fig. 2:**
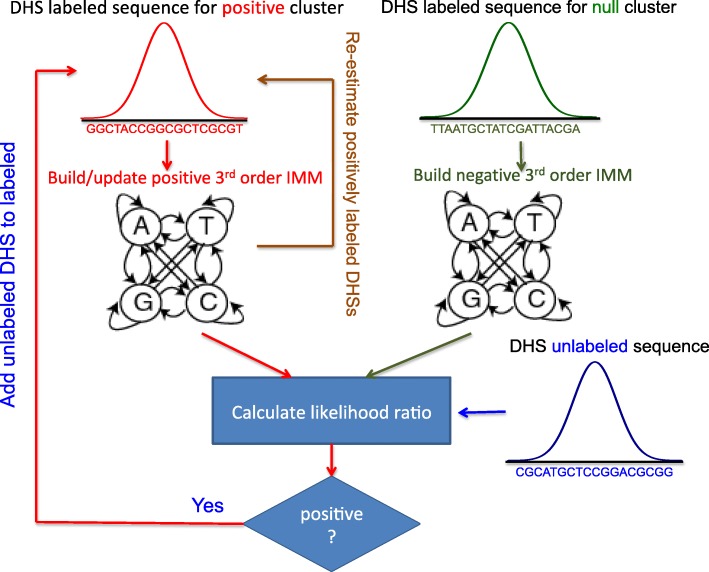
Schematic representation of McEnhancer. Starting with known DHS-gene pairs assigned to a given cluster, it builds a third-order IMM to represent sequence features in DHSs for the positive cluster *(colored red)*. It builds another IMM to represent the null model. In iterative rounds, the model loops on all unlabeled DHSs, calculates log likelihood ratio, and assigns a class label to each unlabeled DHS accordingly. After it finishes looping on all unlabeled DHSs, it adds the newly assigned positive DHSs to the positive cluster and re-estimates IMM parameters. Log likelihood ratios for positively labeled DHSs are re-calculated and re-added to the unlabeled set if their likelihood is low. The model iterates until it converges

To investigate the practical feasibility and performance of such a strategy, we here propose a simpler approach dubbed McEnhancer, which also allows us to utilize the available information on enhancer-gene assignments. As a central part of our approach, McEnhancer learns relevant common subsequences from an initial set of known (labeled) DHS-gene pairs, then predicts assignments for other unlabeled DHSs with similar subsequences for one expression pattern at a time. This is implemented using a third-order interpolated Markov chain model (IMM) in a semi-supervised learning setup via the expectation maximization (EM) algorithm. Due to the very limited number of known DHS-gene pairs to initialize the models, IMMs should be suitable to avoid overfitting. Figure 2 presents a schematic overview of the model.

In a second step, to compare the accuracy against baseline approaches such as assigning enhancers to the closest genes, sparse k-mer-based logistic regression classifiers are then trained on McEnhancer-identified sets of enhancers to predict expression patterns. Coefficients of these k-mers are then used to highlight enriched sequence patterns that might correspond to TF binding motifs. Pseudocode for McEnhancer is presented in Additional file 1: Figure S1, and details on the model setup, parameter selection, and cross validation are provided in Additional file 2.

## Results

### Mapping enhancer candidates from DNase-seq data

**Fig. 3:**
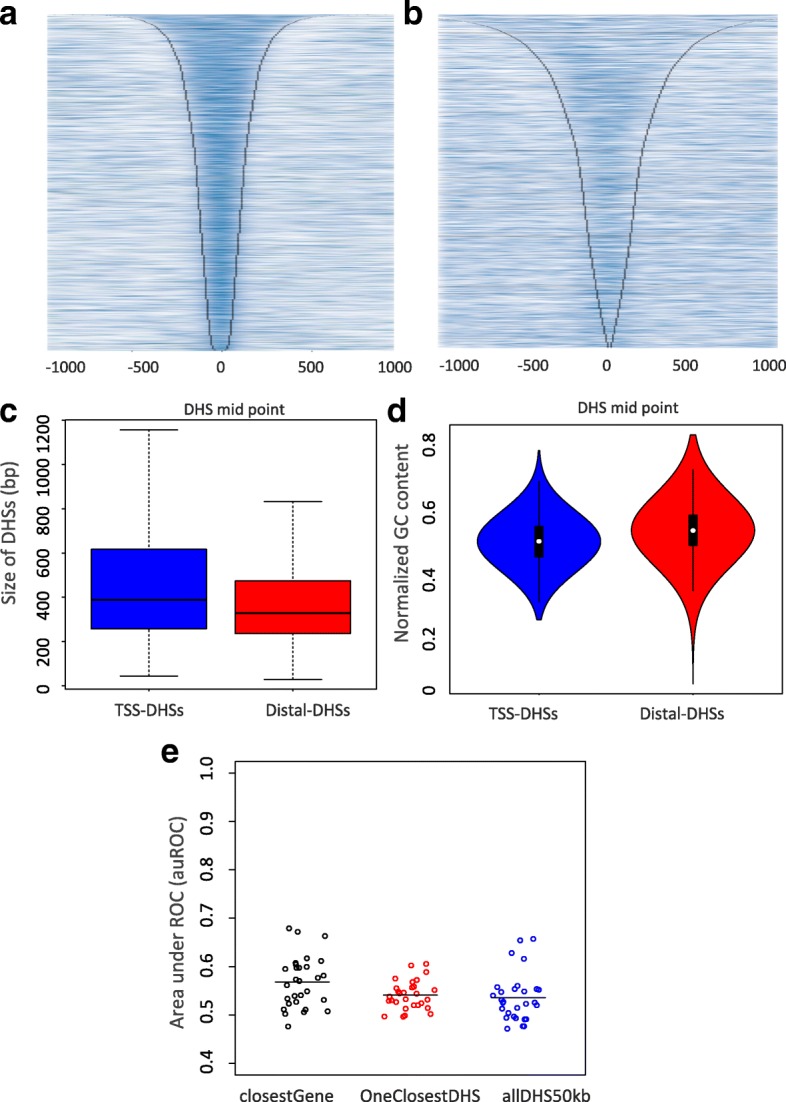
Mapping enhancer candidates from DNase-seq data and baseline classifications. **a** JAMM-identified peaks for DHSs in stage 14, versus (**b**) originally processed DHSs in stage 14. Heatmaps are centered on DHS midpoint, ranked by peak width and show extended read count intensity. **c** Size of all DHSs that overlap TSSs and those overlapping other regions along the genome. **d** Normalized GC content per each group of DHSs. **e** Area under receiver operating characteristic curve *(AuROC)* for classification of various simple baseline methods for distal DHS-gene assignment

Accessibility to DNA modifying enzymes such as MNase and DNase is a generally accepted hallmark of active regulatory regions [13, 36, 40]. DHSs were previously collected from *Drosophila melanogaster* embryos at time intervals centering on stages 5, 9, 10, 11, and 14, respectively [37]. The collection of DHSs across all stages was shown to cover 6.4% of the euchromatic genome (7.6 Mb), with an average of 3.5% at any given stage. Overlapping the published DHSs with previously annotated CRMs from REDfly showed an overlap of 4077 DHSs. This overlap decreased to 2375 DHSs when overlapping published DHSs with CRMs from the VT library. Due to the low signal-to-noise ratio of the processed published DNase peaks (Fig. 3 b), we decided to re-map the raw data and call peaks using JAMM [41]. The overlap of the newly called DHSs was 4111 and 2784 DHSs when intersecting with REDfly or the VT library, respectively. The heatmap in Fig. 3 a shows the improvement of JAMM-identified DHS peaks against the original mapped data for stage 14 of Fig. 3 b (see Methods). These DHSs exhibit marginal differences according to their genomic regions: DHSs overlapping TSSs are a bit larger (median size ∼400 bp), while distal DHSs are smaller (median size ∼330 bp (Fig. 3 c). Distal DHSs show marginally higher GC content than TSS-DHSs (Fig. 3 d).

### Assigning enhancers to their closest genes leads to relatively poor results

In the absence of complementary data, the most common technique to assign enhancers to their target genes is by linking them to the nearest TSS. As a baseline, Distal DHSs were assigned to their closest genes. Then, a sparse logistic regression classifier was used to test the accuracy of this assignment (Fig. 3 e, black dots). Each of the dots represents the area under receiver operating curve (AuROC) for classification of one gene cluster (from the 29 clusters with restricted expression) against ubiquitous genes (10 broad clusters grouped together as the negative set). The average AuROC of ∼0.57 for this assignment is close to random. This is markedly worse than previously indicated in human [13], where the larger intergenic space may allow for more meaningful assignments based on distance.

Another alternative is to assume that each gene is regulated by its single closest DHS. Training classifiers on this smaller subset of DHSs leads to an average AuROC for this assignment that is again almost random ∼54% (Fig. 3 e, red dots). Finally, when all distal DHSs in the region +/- 50 kb around each gene are associated with a corresponding gene, the classification AuROC against ubiquitous genes is ∼53% (Fig. 3 e, blue dots). This implies that all of these baseline enhancer-gene assignment methods are not effective. A smarter model that can link DHSs to their target genes is needed for an understanding of gene regulation specificity.

### McEnhancer links enhancers to their target genes

Rather than assigning a fixed subset or all candidates in a region, we would ideally infer whether any given DHS in a genomic locus would be more likely involved in the regulation of a target gene at the center of the locus; i.e., labels for each distal DHS are hidden variables but may be inferred. McEnhancer was therefore applied to predict a yes/no label for each distal DHS, implying whether that DHS is regulating a given gene. The model is built across all genes with the same expression pattern (here, belonging to one of 17 embryonic restricted expression clusters). Only 17 clusters were used instead of the 29 restricted clusters due to data availability for initialization (see Methods). Simultaneously, McEnhancer identifies a subset of enhancers and their relevant sequence features via multiple iterations in an EM approach.

Known DHS-gene pairs were used for model initialization (numbers shown in Additional file 1: Figure S2); then McEnhancer was run, until convergence, in a pairwise manner on every possible combination, comparing one cluster against each of the other clusters. The predicted DHSs for each cluster from all of its pairwise runs were tallied up, and those above a certain threshold were considered to regulate the corresponding expression pattern. A detailed description of the model is supplied in Additional file 2, and the final predictions for each cluster are given in Additional file 3.

In order to allow McEnhancer to learn the best sequences specific for each gene cluster, we ran McEnhancer in two phases. Phase I targets genes with unique expression patterns (assigned to exactly one cluster). In this phase, DHSs overlapping REDfly CRMs and VTs were used for initialization. In the next step, phase II of the model uses predicted distal DHSs from phase I to predict matching enhancers regulating non-unique genes (i.e. genes assigned to multiple expression classes).

**Fig. 4:**
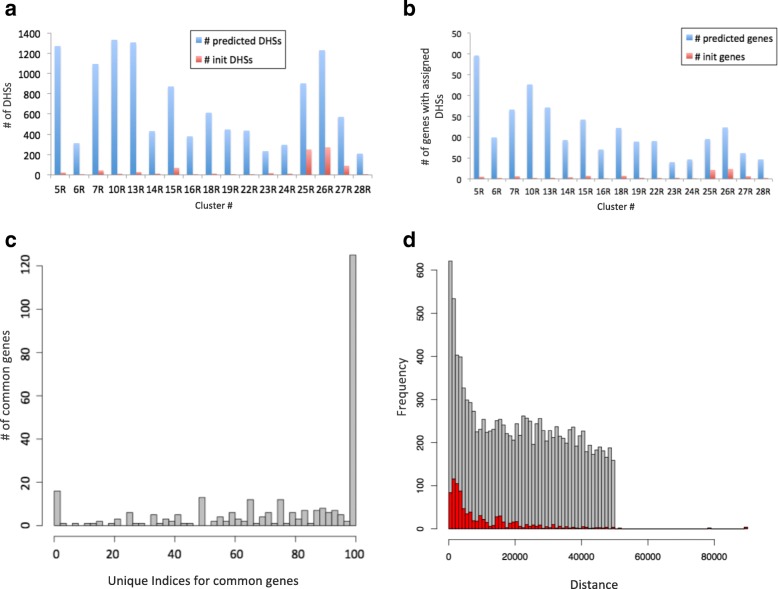
McEnhancer results. **a** Number of unique distal DHSs used in initialization and after prediction. **b** Number of unique genes used in initialization and after prediction. **c** Differential DHS usage among genes belonging to more than one expression cluster. For each of the common genes, the unique index *(UI)* is computed measuring the percentage of uniquely predicted DHSs. A histogram of UIs for all common genes is displayed. **d** A histogram showing the frequencies of distances between predicted DHSs and their corresponding genes. *Gray bars* display distances between predicted distal DHSs and their corresponding genes, while *red bars* represent distances for distal DHSs that were still kept in the final predictions of those used in initialization

Figure 4 a shows the number of unique distal DHSs that were used in initialization, as well as the total number of unique distal DHSs that were predicted for each expression cluster. Figure 4 b shows the number of genes with associated distal DHSs before and after learning for each of the two groups. The difference between the small number of genes with known assigned distal DHSs in the initial start set compared to hundreds of genes with predicted distal DHSs after running McEnhancer is quite remarkable. In total, McEnhancer predicted 9180 unique distal DHSs regulating 1621 unique genes. On average, each patterned gene is thus regulated by 5–6 different distal DHSs. Importantly, only 23% of predicted distal DHSs were assigned to their closest genes, with the rest being assigned to more distal ones.

The results so far were based on an initialization in which we combined two datasets of enhancer-gene pairs, namely REDfly and VT libraries. To assess the robustness of enhancer selection and validate the accuracy of the selected DHSs, we now initialized with one of the two datasets only (REDfly), leaving the other one (VT) for an independent validation of McEnhancer across all clusters (see Methods). In this setting, McEnhancer, after phase I, correctly predicted an average of ∼90% of the enhancers from the VT set that were assigned to each cluster (Additional file 1: Figure S4-A). Overall, this quantifies the robustness of McEnhancer to recover known enhancer-gene assignments on genomic datasets. This also allowed us to rule out a possible bias for distal interactions. Additional file 1: Figure S4-B shows the distribution of enhancer-gene distances in the VT set, and in the recovered subset when initializing on REDfly only.

### Distances between enhancers and their associated genes span large ranges

We next investigated the distances between predicted distal DHSs and their corresponding genes (Fig. 4 d). There is a preference for distal DHSs to regulate genes within closer distances, represented through the high peak at shorter distances. However, it flattens out with increasing distance and becomes uniform across the search space window, +/–50 kb, around the genes’ promoters. A previous model presented by [25] showed that enhancers that are 50 kb away from genes’ promoters were predicted to influence their associated genes.

Setting a hard limit of a 50kb window around genes promoters may therefore have been a tight constraint. However, if multiple genes exist in the +/–50-kb region that belong to the same gene cluster, all of these genes will be assigned to the same enhancer with the same probability. Additional file 1: Figure S3 shows that the total number of such genes falling within 100 kb of each other is approximately 11% per cluster. (The few distal DHSs with distances larger than 50 kb result from the initial labeling.) Increasing this window would also increase the number of genes belonging to the same cluster that fall within the same window. In compact genomes, this conundrum can effectively only be solved by using data for direct interactions.

### Genes with multiple expression patterns are regulated by distinct sets of enhancers

Apart from the distance, we examined whether genes belonging to multiple expression clusters would be predicted to be regulated by different enhancers. We calculated a uniqueness index (UI) for each gene belonging to multiple expression clusters (see Methods). Frequencies of differential DHS usage are shown (Fig. 4 c). It is interesting to see that almost half of the genes that belong to more than one expression cluster are regulated by completely different sets of DHSs (marked by the long bar at 100%). This indicates that genes belonging to multiple expression patterns are predicted to be regulated by different modularly acting enhancers.

### Temporal patterns of predicted DHSs match gene expression time points of assigned clusters

**Fig. 5:**
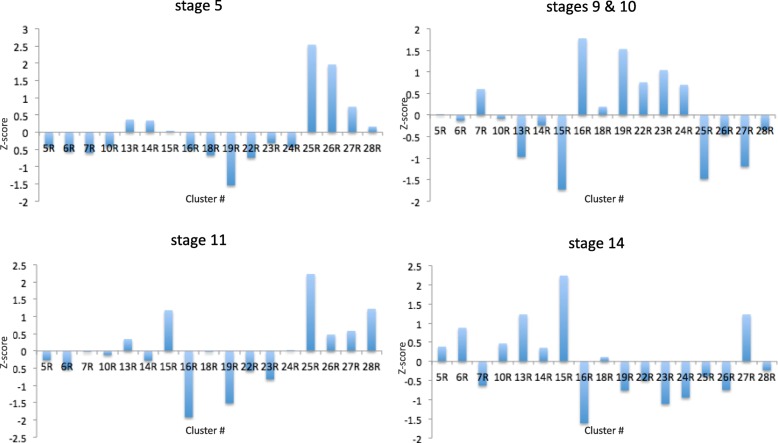
Stage enrichments in predicted DHSs. Predicted DHSs are enriched for developmental stages corresponding to expression patterns of genes in corresponding clusters

To test whether predicted DHSs match the same developmental stages as that of the associated expression pattern, we counted the number of DHSs predicted in each stage per cluster. Normalized z-scores were then calculated for each developmental stage (Fig. 5). For example, DHSs linked to blastoderm gene expression clusters, clusters 25R, 26R, and 27R, exhibited higher accessibility than other gene clusters in early accessibility data (higher z-scores in stage 5). Importantly, genes of these clusters are highly expressed in the early stages of development (around stage 5), which is in concordance with increased accessibility of active enhancers driving the activity of these genes. Similarly, clusters with nervous system expression, such as clusters 13R, 14R, and 15R, have high accessibility z-scores for later (stages 11 and 14). This complies with expression of their genes, where they are mainly expressed in stages 11, 12 and 13–16.

### Active enhancer-associated histone marks are enriched at predicted enhancers

To validate that McEnhancer selects functional enhancers, we first analyzed the chromatin structure in the vicinity of the selected regions for hallmarks of enhancer activity. Ideally, the data on chromatin structure (e.g., histone modifications) were to match the cell type in which the enhancers are active. We therefore utilized mesoderm-specific histone modification ChIP-seq data for two enhancer-related histone marks, H3K4me1 and H3K27ac [8], and visualized peak coverage on selected DHSs for clusters 18R and 19R (genes expressed in differentiated somatic muscle and differentiated visceral muscle; see Methods).

**Fig. 6:**
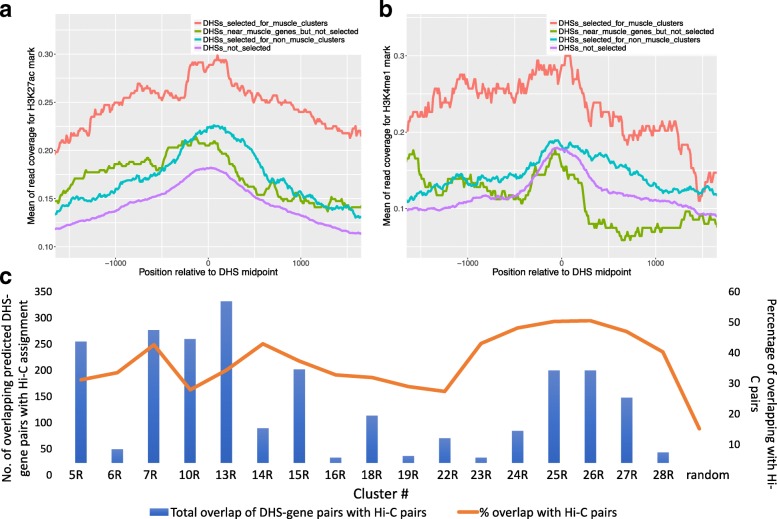
McEnhancer predictions are validated through enrichment of histone marks and overlap with Hi-C data. Enrichment of histone modification marks: **a** H3K27ac and **b** H3K4me1 for clusters with predicted mesodermal expression (red), compared to DHSs near these mesodermal genes but were not selected by McEnhancer (green), DHSs selected by McEnahncer but with no mesodermal activity (blue), and DHSs that were not slected to regulated any of the clusters (purple). **c** Validation of predicted DHS-gene pairs by overlapping with identified fragment-promoter Hi-C interactions. For each gene cluster, number of DHS-gene pairs which overlap with Hi-C fragments and for which these fragments were linked to the same genes was counted (blue bars). Percentages represented by this overlap with respect to filtered predicted DHS-gene pairs are shown by the orange line. Random refers to null expectation derived from data shuffling, see Methods

Figure 6 a shows enrichment of H3K27ac, a chromatin modification indicative of active enhancers, at DHSs selected for clusters with predicted mesodermal expression. We compared this pattern to DHSs that were near these mesodermal genes but were not selected by McEnhancer, DHSs selected by McEnhancer with non-mesodermal enhancer activity, and DHSs not selected to regulate any of the clusters. Similar to H3K27ac, we also observed higher levels of H3K4me1 on predicted tissue-specific enhancers in tissue-matched chromatin data; see Fig. 6 b. This underscores the predictive power of McEnhancer and supports the argument that H3K4me1 is elevated at active enhancers compared to non-active ones [42]. Furthermore, Additional file 1: Figure S5-A highlights the fact that enrichment of these histone marks is not simply correlated with DHS accessibility. Together, these observations support our prediction that these enhancers are indeed regulatory sequences active in mesoderm-specific tissues.

### Identified Hi-C fragment-promoter interactions validate the DHS-gene predictions

In addition to histone modification enrichments that support the condition-specific enhancer function, direct evidence for enhancer-promoter interactions may be extracted from chromatin conformation data such as Hi-C. Given its relatively low resolution and experimental bias, Hi-C data needs to be processed by tailored analysis pipelines aimed at validating enhancer-gene assignments. Therefore, previously published genome-wide Hi-C raw data from *Drosophila* embryonic nuclei [43] was processed with the High-throughput Identification Pipeline for Promoter Interacting Enhancer elements (HIPPIE), which specifically calls significant Hi-C interactions between promoters and distal locations [44]. In this way, on average, ∼40% of DHS-gene pair predictions are confirmed by exact fragment-promoter assignment (Fig. 6 c). To assess the significance of this overlap, we derived a null distribution from random permutations of the data, while correcting for the enhancer-gene distance skewness (Additional file 1: Figure S5-B; see Methods).

### McEnhancer learns specific regulatory sequences for each expression pattern

**Fig. 7:**
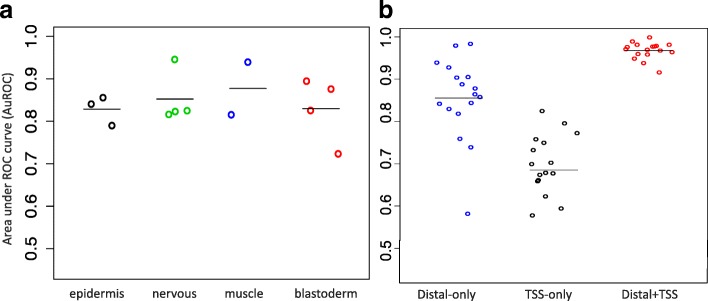
McEnhancer learns specific patterns for each expression pattern. **a** Sparse logistic regression classifier for each restricted expression cluster against distal DHSs assigned to all other clusters. For better visualization, clusters are grouped by their general biological function annotation, as defined by [30]. Classification of clusters with less-defined functional annotation also gave similar results, results not shown. **b** Sparse classification for each cluster against ubiquitous genes for promoter features alone and with addition of promoter to distal features as separate vector

McEnhancer assigns enhancers to their genes, based on probabilistic sequence models, in an individual manner. This does not guarantee that a gene’s expression pattern can be predicted from enhancers that were assigned to each gene. To measure the accuracy of McEnhancer in assigning distal DHSs to restricted expression clusters, we used sparse logistic regression classifiers trained on the selected enhancers to quantify the extent to which prediction of expression patterns improved. For genes from a specific cluster with restricted expression, the assigned distal DHSs were classified against the distal DHSs assigned to all other clusters with restricted expression. The classification performance for each class is displayed in Fig. 7 a, with an AuROC average ranging from 80 to 90%. This high classification performance reflects the ability of McEnhancer in learning distinct sequence features for each gene’s expression pattern, and quantifies how much better the prediction of gene expression patterns is, across all genes and all enhancers selected, compared to the baseline models above.

### Regulatory sequence information in proximal promoter DHSs sharpens the specificity of expression pattern prediction

McEnhancer is only concerned with distal DHSs; promoter DHSs are assumed to regulate their closest genes. To examine the effect of regulatory features in promoter regions in dictating the specific expression pattern of the cluster, DHSs overlapping gene TSSs in each expression cluster were classified against those overlapping TSSs of ubiquitous genes. Classification resulted in an average AuROC ∼68% (Fig. 7 b, black dots). Adding promoter DHSs to distal DHSs, each as a separate feature vector, resulted in a high AuROC ∼97% (Fig. 7 b, red dots). This implies that information retained in promoter regions is not enough to encode specific expression patterns; however, promoters together with the McEnhancer-assigned distal enhancers allow for an almost perfect distinction between different expression patterns.

### Functional validation of predicted enhancer activities for identified DHSs

To assess whether our strategy to identify enhancers by chromatin accessibility and to assign them to target genes holds true in vivo, we aimed to test several putative enhancers in developing embryos. However, because we assign DHSs to putative target genes via concordance of their k-mer content with that of verified enhancers linked to target genes of known expression classes, a first indication of the quality of predicted enhancer-target linkages is k-mer content itself — do the DHSs assigned to the specific expression classes contain sequence motifs for TFs likely to regulate the respective expression patterns?

#### DHS classes are enriched for sequence motifs for TFs known to regulate the linked expression patterns

We analyzed DHSs assigned to each expression pattern for statistical enrichment of sequence motifs (5-mers) and assigned them to TFs based on reported binding preferences using the Analysis of Motif Enrichment (AME) algorithm [45]. AME computes the statistical enrichment of known motifs in a set of sequences versus other control sequences based on threshold-free linear regression (see Methods). TF assignment is often ambiguous because TFs can bind to a range of sequences, TF complexes (e.g., dimerization partners) may alter sequence preference, and several TFs may be assigned to the same motif. Furthermore, as we use sparse classifiers, they will only use a subset of possibly informative k-mers, and well-known factors may be absent. Despite these caveats, we could assign many TFs that explain much of the expression behavior of the respective clusters (Additional file 1: Figure S6, summarized in Additional file 4).

For example, cluster 18R genes are expressed primarily in the somatic mesoderm (SM), and associated DHSs were enriched in motifs assigned to the Myocyte Enhancing Factor 2 (*Mef2*), which plays a key role in SM differentiation (see, e.g., [12]). Similarly, another enriched motif maps to sine oculis (*so*), which is a homeodomain TF related to Six4. *so* is unlikely to play a role in SM development, but *Six4* has a very similar binding motif [46], is expressed in the mesoderm at the appropriate stage, and has been shown to be a key patterning agent of the ventral mesoderm, from which the SM derives [47].

Cluster 19R genes are primarily expressed in the visceral mesoderm (VM). The enriched motifs include 5-mers matching the TF tramtrack (*ttk*), which has recently been found to play an important role in cell fate specification in the developing mesoderm and especially the VM [48], as well as motifs matching *nautilus*, which is involved in myogenesis. Interestingly, a motif for the well-known repressor *snail* [49, 50] is also enriched in 19R. Snail is expressed in the presumptive mesoderm early, but exclusively in neurogenic tissues starting at stage 10. This implies a temporal mechanism to achieve tissue-specific expression — Snail may keep genes off first in the presumptive mesoderm and later in the neurogenic tissues, while allowing their expression in the VM. Similarly, the Zn-finger TF *abrupt* is a putative new negative VM regulator; *abrupt* is not expressed in the mesoderm but may serve to repress VM genes in other tissues. Another enriched motif corresponds to ETS binding motifs. ETS transcriptional activators like *pnt* require ERK signaling, which is activated in the VM [51] — it is feasible that ETS sites act like a bimodal switch, where ETS TFBSs allow enhancer activity in the VM via activated Pnt, but deny activity of the same enhancers via the ubiquitous ETS repressor Aop elsewhere.

Similarly, motifs matching known neurogenic TFs are found in nervous system clusters 15R and 16R, and various early patterning determinants are found in blastoderm clusters 23R–27R. However, it should be noted that the expression classes are not homogeneous. Rather, they are complex aggregates relying on diverse and distinct regulatory logics [30]. While speculative to a certain extent, the fact that functional motifs can nonetheless be distilled is encouraging. More finely grained expression data would certainly enhance motif identification, TF assignment, and target gene prediction, especially as tissues become more complex.

#### In vivo activity of DHSs indicates them to be enhancers of the predicted reporter genes

To assess enhancer activity of identified DHSs and to validate their target gene predictions, we co-visualized DHS-directed reporter and predicted target gene expression in transgenic embryos. A previous study identified thousands of putative mesodermal *Drosophila* enhancers based on TF binding assays [12], but it focused on enhancer activity and made no attempt to predict target genes. We randomly chose 5 of the reported enhancer lines that overlapped with DHSs and examined gene expression.

**Fig. 8:**
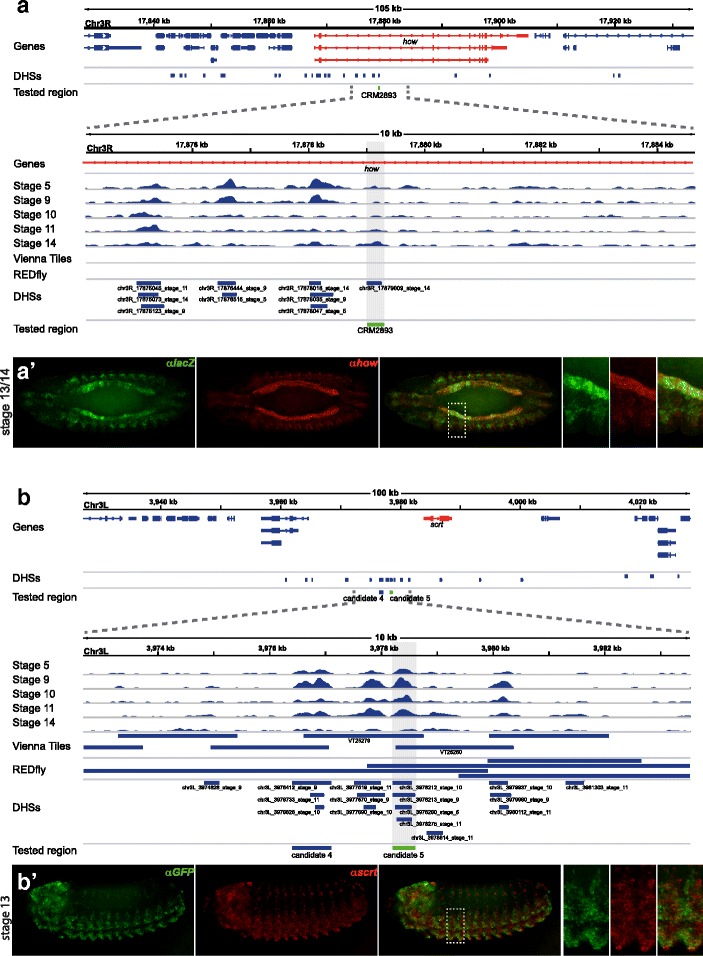
DHSs tested in vivo direct gene activity similar to predicted target genes. Shown are the genomic environments for the tested putative enhancers **a** CRM2893 and **b** candidate 5. Gene models, Vienna Tiles and DHSs are shown (blue), predicted target genes are red, tested regions are green. DNA accessibility at 5 developmental stages is shown across 10kb regions below. **a’** and **b’** show reporter gene activity directed by the enhancer candidate (green) and the expression pattern of the predicted target gene (red). Shown is a medial cross section, ventral view (**a**) and a ventrolateral view (**b**), anterior left; stippled boxes outline the zoomed-in regions on the right
- The DHS covering a second mesodermal enhancer, CRM6053, also appears to be a functional and correctly assigned enhancer and drives much of the expression pattern of its target gene *noc* from stages 8–11 (Additional file 1: Figure S7-A).
- A third DHS matching CRM5481 is accessible at stages 11–14 (Additional file 1: Figure S7-B) and was predicted to target *tkv* and *Bsg25D*. The DHS directs expression in the dorsal mesoderm (stage 11/12), SM (stage 13), and anterior VM patches (stage 15) (Additional file 1: Figure S7-B). *tkv* expression nicely co-localizes with reporter gene expression in all three mesodermal tissues (Additional file 1: Figure S7-B), whereas *Bsg25D* is co-expressed only in the SM [52]. Why these two genes may be targeted tissue-specifically by the enhancer DHS is unclear, but note that while the DHS is intronic to *tkv*, it needs to act over 25 kb to target the *Bsg25D* promoter. Nonetheless, it seems that this DHS enhancer has been correctly mapped to both target genes.
- The fourth mesodermal enhancer DHS, CRM3775, drives expression in the mesoderm primordium at stage 5 and in the SM at later stages, which is in agreement with accessibility data (Additional file 1: Figure S7-C). One of the two predicted target genes, *CG2162*, does not significantly overlap with reporter expression, but target *CG32486* shows expression very similar to that of the reporter gene in areas surrounding hindgut and foregut invagination at stage 8, as well as in the SM after stage 13 (Additional file 1: Figure S7-C). Therefore, the DHS seems to have been correctly matched to one of its two predicted targets.
- The fifth DHS we tested matched CRM4515, is accessible continuously, and was predicted to target three genes: *HGTX*, *bbg*, and *CG9238*. None of the target genes is expressed in domains overlapping CRM4515 activity (Additional file 1: Figure S7-D [52, 53]), but it should be noted that reporter and predicted target gene expression are extremely complementary (e.g., *HGTX*, see Additional file 1: Figure S7-D), which in this case suggests that the regulatory information contained within the DHS serves to activate transcription of the reporter gene in the transgenic context but to mediate repression in the endogenous context, possibly due to CRM truncation.

- The transgenic line CRM2893 was reported as a visceral mesoderm (VM) and somatic mesoderm (SM) enhancer at developmental stages 12–16. Temporal DHS accessibility matches reported enhancer activity (Fig. 8 a), and the DHS was predicted to target the gene *how*. Importantly, we find near-perfect overlap between *how* and CRM2893-directed reporter gene expression in the VM and pharyngeal muscle (Fig. 8 a’), as well as significant overlap in the SM (not shown). We conclude that this DHS constitutes a developmental enhancer and was correctly mapped to its target gene.

Additionally, we investigated several enhancer predictions linked to the neurogenic expression cluster. Two of these candidates (4 and 5) are located within 1 kb of each other and may be part of the same larger regulatory region. While candidate 4 directs no detectable reporter gene expression on its own (not shown), candidate 5 directs expression in the ventral nervous system primordium (Fig. 8 b). The overlap of reporter gene activity with expression of the predicted target gene *scrt* indicates correct target assignment for candidate 5 (Fig. 8 b).

Candidate 1 was predicted to target *soxN* at a distance of more than 20 kb, but showed no detectable regulatory activity. Candidate 3 is primarily accessible at stage 5, which is when it directs ventral expression in a stripe-modulated pattern and ectodermal patches later. The predicted target genes are *nuf* and *Dichaete*, but while no reporter overlap with *nuf* expression was detected, some limited expression overlap was observed for *Dichaete* anteriorly at stage 5, as well as in the ectodermal patches later (Additional file 1: Figure S7-E).

Though candidate 3 may well regulate aspects of *Dichaete* expression, the limited overlap of reporter gene and target gene expression sometimes observed (e.g., *Dichaete*, *noc*) highlights that many genes are likely to be regulated by the interplay of several modular enhancers. While we have observed cases of extremely close correspondence between enhancer activity encoded in a DHS and the expression of the predicted target gene (e.g., *how*, *noc*, *tkv*, *scrt*) — underscoring McEnhancer accuracy — some cases stand out where the prediction was seemingly inaccurate. In cases such as *nuf* and *CG2162*, this is likely due to insufficient resolution in expression classes — both genes are marked as blastoderm expressed, and while both DHSs drive blastoderm expression, the specific patterns are distinct. Better expression class resolution would likely remedy even these cases.

Taken together, our results show that DHSs not only serve as a valuable tool for the identification of likely regulatory elements, but that given an appropriate training set (i.e., linked enhancers and reliable gene expression classification), their enhancer activities can be predicted globally, and they can be assigned to putative target genes with considerable accuracy.

### Verification of long-range interactions

Finally, we evaluated the accuracy of McEnhancer-target gene associations with respect to some challenging enhancer-target assignments, specifically cases where the experimentally determined target gene is not the closest gene. The REDfly database [38] contains 62 such instances, of which 26 uniquely overlap with our DHSs, 14 assigned to genes with unique expression pattern, and 12 assigned to genes with multiple expression patterns; the latter 12 cases were not used in initialization (see Additional file 2) and thus constitute an independent test set. Out of those, the McEnhancer-target predictions directly agree in 9 cases (75%). Upon taking a closer look at the reported enhancer activity compared to that of the McEnhancer assigned gene, we determined that the accuracy of assignment is possibly as high as 11 or 12/12 (92–100%). Two of the three questionable cases seem to be in fact correctly assigned by McEnhancer (see Additional file 5): in one case, the CRM’s activity clearly overlaps with the expression of the assigned gene, in the other it appears that not only do enhancer activity and target gene expression match, but the target gene assigned by McEnhancer is indicated by the original publication [53] as a likely target and was simply incompletely annotated in the REDfly database. The final case is inconclusive: the enhancer activity of another DHS (accessible at >stage 9) likely matches the reported weak expression of the predicted target gene *(CG3838)* in the ventral nerve cord [52]; although no images of the CRM’s activity are available, its activity is described as “st. 9-16 lateral epidermis (weak), CNS (weak)” [54]. Note that in the 26 cases (Additional file 5), the simple closest-gene assignment would have failed.

## Discussion

We have approached the general problem of predicting gene expression from sequence, by linking enhancers to putative target genes by means of identifying a subset of enhancers with similar sequence composition/k-mer counts around co-regulated genes. With 62,453 DHSs, rich in putative enhancers, 13,659 protein-coding genes, and only a few known DHS-gene pairs, predicting enhancer-gene pairing globally becomes a complex machine learning and data analysis challenge. DHSs were used to represent potential candidates of transcriptional enhancers. Since enhancers are believed to be relatively “open” when regulating their target gene(s), McEnhancer only searches in DHSs as proxy for putative enhancers instead of the whole genome. Since we combined DHSs across several embryonic stages, this certainly implies that many of these DHSs are irrelevant for establishing the considered temporally resolved expression patterns, but the process decreased the search space to 6.4% of the euchromatic *D. melanogaster* genome.

Together with clusters of genes expressed in similar patterns in embryonic development, a semi-supervised machine learning model was built to predict linkage between DHSs and their associated target genes. For a given gene cluster, McEnhancer first builds a third-order interpolated Markov model on small starting sets of known target gene assigned regulatory regions documented in REDfly and the VT library. Through application of the EM algorithm within a pairwise semi-supervised learning framework, it then scores each of the unlabeled DHSs in a +/– 50kb window around genes in the cluster. Labeled and unlabeled sets are updated accordingly, and the whole model parameters are re-estimated based on the newly updated sets. In this work, using interpolated Markov chains is crucial to avoid overfitting. The algorithm iterates until convergence, and DHSs predicted to harbor regulatory information associated with specific expression clusters are identified.

Predicting gene expression from enhancer candidates has previously worked to a certain degree even when assuming that enhancers regulate their closest target [13, 39]. We could show that the prediction of expression patterns increased dramatically after selection of a subset of enhancer candidates via McEnhancer. The high performance of logistic regression classification between predicted DHSs for each specific cluster against other clusters, as well as against ubiquitous enhancers, supported the notion that we selected a subset of enhancers relevant for the expression of the target genes. This observation was also corroborated by the match between developmental stages of selected DHSs and expression time points of their associated genes. While our approach provides a two-step solution to first select enhancers and then build classifiers, it is conceivable to phrase the problem within an integrated probabilistic model that explicitly treats the enhancer-gene assignments as missing/hidden variables.

Assigning enhancers to the gene with the closest TSS appeared to be a comparatively poor assumption for the compact genome of *Drosophila melanogaster*, where genes are close to each other, yet enhancers have been shown to influence regulation over large genomic distances. Our analysis shows that only 23% of predicted DHSs are assigned to their closest genes. This number may be somewhat low, as there may be cases where the closest gene regulated by an enhancer was not part of the pre-defined expression clusters, or where an enhancer regulates its closest target gene, but in a different expression context not considered here. However, other recent studies have also reported that enhancers regulate surprisingly few of their closest genes. For example, a study analyzing 5C data in human showed that only 27% of distal elements have an interaction with their nearest TSSs [27]. Similar results were also obtained from another study analyzing ChIP-seq data in mouse [28]. Reconstruction of gene regulatory networks suggests that sometimes more distal regulatory elements control gene expression over those that are positioned closer to the gene [24]. Thus, when predicting enhancer-gene interactions, choosing the nearest gene may be globally informative, but insufficient and misleading in specific cases. Furthermore, as with all approaches that do not use evidence of direct interaction data, we cannot resolve cases in which multiple co-expressed genes are located in the same genomic neighborhood (here, several genes from the same cluster within a +/– 50kb region). Using topologically associating domains (TADs) instead of fixed-sized windows may resolve some of such cases, and it is in fact a matter of current debate whether co-expressed genes within a TAD are generally differentially or jointly regulated by shared enhancers.

The presence of enhancer-related histone modifications in the vicinity of enhancers is generally used to validate predicted enhancers. Enrichment of H3K27ac in predicted enhancers revealed active regulatory function in both *Drosophila* and human embryonic cells [7, 20, 55]. The existence of H3K4me1 marks at predicted enhancers adds an extra layer of validation, as H3K4me1 has been described as a chromatin mark associated with enhancers irrespective of activity [56].

Analyzing genome-wide Hi-C *Drosophila* embryonic data [43] using HIPPIE [44] showed a considerable overlap of identified fragment-promoter interactions with predicted DHS-gene pairs. Here, the available read depth and the compactness of the fly genome allowed for a meaningful analysis at single gene loci, which is not yet feasible for most if not all available mammalian datasets; yet, it is important to note that due to read coverage bias and the current resolution of Hi-C data, the lack of interaction between two fragments in Hi-C data analysis does not rule out the existence of the interaction.

Investigating the k-mer sequences that contributed most to the expression pattern classifiers provided deeper insights into specific gene regulation. k-mers to which non-zero weights were assigned by the sparse logistic regression were mapped to TF binding preferences to identify candidate regulators. Examples of meaningful matches derived in this way included motifs known to be able to recruit crucial regulators directing expression in and shaping the developmental trajectories of the respective expression domains.

Such analyses are necessarily incomplete and require careful interpretation as discussed, but are nevertheless useful in highlighting enriched motifs that offer potential TF candidates for regulating a specific system for further validation. Finally, we were able to test and verify the regulatory activities of several identified DHSs. Of nine tested DHSs, seven were found to act as enhancers and were able to direct reporter gene expression in vivo. Of these, six exhibited partial overlap with the expression of predicted target genes; the seventh was driving expression in a pattern curiously complementary to that of all three predicted target genes. In some cases, the DHS captured the regulatory activity explaining a target gene’s developmental expression pattern almost entirely. This indicates that not only are we able to predict enhancers based on DHSs, but that McEnhancer is also able to assign target genes, and even multiple target genes for a single enhancer, with considerable accuracy, which will further improve with more accurate expression classification, both temporally and spatially.

## Conclusions

This study provides a new approach to the problem of assigning enhancers to the genes they regulate. Via semi-supervised machine learning, we can start from a handful of positive samples and add further unclassified samples around co-expressed genes, which look most similar in terms of their sequence composition. We also posited that successfully predicting specific expression patterns from the assigned enhancers is a highly effective approach in evaluating the success of enhancer-gene assignments. In summary, this approach provides a framework for making sense of large in vivo regulatory datasets that do not completely align with one another and do not have cell-type-specific resolution. It is an example of how machine learning provides an effective means to provide deep insights into the biology of gene regulation, and provides a starting point for improved computational and experimental strategies.

## Methods

### DNaseI data processing and peak calling

In order to identify regions of enriched accessibility in DNaseI, referred to as DNaseI hypersensitive sites (DHSs), peaks were called on raw data. DNaseI raw data for two replicates of *Drosophila melanogaster* embryos at 3, 4, 5, 6, and 11 hours, corresponding to stages 5, 9, 10, 11, and 14 in embryogenic development was downloaded, SRP002474 [37]. Bowtie2 was used to map raw data to the *Drosophila melanogaster* genome (dm3) [57]. After this, peaks were called on each replicate separately using JAMM [41] and the irreproducible discovery rate (IDR) between the two replicates was calculated. Peaks with only IDR ≤ 0.02 (2% threshold) were considered. This resulted in a total of 62,453 DHS peaks for all stages combined.

Heatmaps showing JAMM-identified DHS peaks in stage 14 are represented in Fig. 3 a. These heatmaps are centered on peak center (DHS midpoint) and ranked by peak width. Corresponding peak edges are shown by gray lines. When compared against the original processed signal, which uses a scan-statistic algorithm to identify DHSs (Fig. 3 b), the JAMM peaks are better identified and their signal-to-noise ratio is higher.

### Assembling known DHS-gene pairs (labeled data)

The gold standard for testing enhancers and their associated genes is through designed reporter assays. In these experiments, a candidate DNA sequence is placed upstream of a minimal promoter and a reporter gene. The activity of the enhancer is then measured by the abundance and localization of the reporter transcript, or the reporter gene is detected by enzymatic activities, fluorescence, or specific antibodies [10]. These experiments are very low throughput; they are designed to test exactly one specific enhancer against one gene. The broadest and most comprehensive available resource for curated experimentally verified fly CRMs along with their associated genes is the Regulatory Element Database for *Drosophila* (REDfly) [38].

Distal DHSs were overlapped with known CRMs from REDfly (v3.0) and split into expression clusters according to their associated genes. The number of known DHS-gene pairs overlapping REDfly CRMs per each gene expression cluster is shown in Additional file 1: Figure S2 (red bars). It is clear that the number of known DHS-gene pairs is fairly small in most gene expression clusters, with some clusters having not even a single known pair.

Another set of known enhancer-gene pairs is in vivo validated through a high throughput enhancer trapping in situ protocol using transgenic fly lines VT and systematically assigned to their targets [39]. In the VT fly library, each line contains a transcriptional reporter construct with a ∼2 kb candidate enhancer, minimal promoter, and GAL4 reporter gene integrated into an identical position in the fly genome. In situ images for each transgenic line were acquired, and the enhancer activity patterns were manually annotated using a controlled vocabulary. Out of 7705 tested candidate enhancers, 46% were active, with most of them showing specific spatial patterns during development. To associate a target gene for each of these enhancers, the expression patterns of the five upstream and downstream neighboring genes were manually inspected and compared to that of the enhancer in consideration. A gene was assigned to a given enhancer if the gene expression pattern matched that of the enhancer. This manual enhancer-gene pattern association analysis was successful in the linking of only 482 enhancers to their target genes. In the case when a VT contains more than one enhancer, all of these enhancers are equally used in initialization. However, in our semi-supervised framework, enhancers are “allowed” to leave the set in each iteration; if an enhancer used in initialization will not be matching the model during learning, it will no longer be included in the positive set.

Distal DHSs were overlapped with the 482 manually associated enhancer-gene pairs, based on their annotated expression patterns from in situ images. The numbers of DHS-gene pairs per each cluster are shown in Additional file 1: Figure S2 (green bars). Since the number of known DHS-gene pairs per each cluster was still small, a combination of known pairs from REDfly and VT was collectively considered. Some of these pairs are exactly the same, while others are different, Additional file 1: Figure S2 (purple bars). Using both sets provided us with sufficient known DHS-gene pairs that were used for model initialization. Gene expression clusters with no known DHS-gene pairs or with ≤ 3 pairs are discarded from the analysis. A total of 17 clusters were then used for prediction.

### Calculating distances between enhancers and their associated genes

Distances are measured between gene TSSs and centers of distal DHSs. If a gene has more than one transcript, the shortest distance is used. Gray bars in Fig. 4 d display distances between predicted distal DHSs and their corresponding genes, while red bars represent distances for distal DHSs that were still kept in the final predictions out of those used in initialization.

### Calculating uniqueness index for common genes

To further investigate if genes belonging to multiple expression clusters are regulated by different enhancers, we calculated a uniqueness index (UI). First, genes used by McEnhancer which belonged to more than one cluster were marked, and 303 genes were referred to as common genes. Then the symmetric difference of the clusters for which a given gene belongs to was calculated for each of these common genes, and referred to as UI. A UI is defined as the percentage of uniquely selected DHSs out of all predicted DHSs for a given gene. Using set theory, UI for a common gene *x* that belongs to two clusters *A* and *B* is defined as:

1 $$\begin{array}{*{20}l} UI_{x} = \frac{(DHS_{A}-DHS_{B}) \cup (DHS_{B}-DHS_{A})}{DHS_{A} \cup DHS_{B}} \end{array} $$

### Enhancer-associated histone marks

A common technique to validate predicted enhancers is to analyze chromatin structure in the vicinity of predicted regions. To this end, mesoderm-specific histone modification (H3K4me1 and H3K27ac) and histone H3 density ChIP-seq data were obtained from [58]. Raw data was mapped to *Drosophila melanogaster* genome build dm3 using Bowtie2 with default parameters. Only reads that aligned uniquely to a single location were retained. PCR duplicates were removed using Picard (http://broadinstitute.github.io/picard). Peaks for the two modifications were called with JAMM [41], setting parameters -e auto, -d y and -r region, using histone H3 density as control data.

Enrichments of the JAMM signal for each of the two histone marks, specifically H3K4me1 and H3K27ac, were compared across four different groups of DHSs. The first group is composed of DHSs that were predicted by McEnhancer to regulate genes with mesodermal expression pattern (clusters 18R and 19R). The second group consists of DHSs that are near mesodermal genes, but were not selected by McEnhancer. The third group represents other predicted DHSs that were selected for other clusters, other than muscle-related ones. And finally the last group included all other DHSs that were not selected to regulate any of the clusters.

To perform this analysis, a normalized read coverage for each merged BAM file, with no duplicate reads, was obtained for each of the three histone marks. Reads were grouped in 10-bp bins along the genome with extended fragment length. Fragment length was computed as the average of the three fragment lengths after mapping each of the different time points separately. Then, for each group of DHSs, the number of reads that overlapped the corresponding regions were summarized, and the mean score for each bin (10 bp) in the interval of +/–2 kb around the center of the DHS was used to indicate the enrichment score. A local version of deepTools was used in this analysis [59].

Nucleosomes in the vicinity of active enhancers typically contain histone characteristics with post-translational modifications. Figure 6 a shows enrichment of the H3K27ac chromatin mark at the four defined sets of DHSs. Predicted enhancers show high levels of H3K27ac, even when compared against those DHSs that were not selected. This indicates that predicted enhancers are indeed active regulatory sequences, since H3K27ac is a critical mark that separates active from poised enhancers [55]. Predicted enhancers also show high levels of H3K4me1, see Fig. 6 b, which marks the location of enhancers.

### Initialization with one exclusive dataset

To computationally validate the selection of DHSs for each cluster, McEnhancer was initialized with DHSs from REDfly only and then tested regarding how many DHSs from VT were correctly assigned. Clusters with less than 4 DHSs for initialization were excluded from this analysis. In addition, two other clusters did not have any assigned DHSs from VT and were also excluded. This left us with 11 clusters to validate. McEnhancer was run in pairwise settings, comparing a given cluster with the other 10 clusters, for stage 1 of the algorithm where only genes that are uniquely assigned to a single cluster were considered. DHSs selected more than five times for a given cluster across all 10 pairwise comparisons were considered to predict the expression pattern of that cluster. Percentages of overlapping matches were calculated based on the full initial set of DHSs that were assigned to each cluster by VT, as well as the subset kept by McEnhancer when training on both REDfly and VT.

### Enhancer-promoter interactions from embryonic Hi-C data

Currently, the most comprehensive pipeline that tackles many of the shortcomings of Hi-C biases is HIPPIE [44, 60]. In this pipeline, raw data is first mapped to the genome without the use of Hi-C pairing information. Because the resolution of Hi-C is constrained by the length distribution of the fragments produced by the chosen restriction enzyme, the reads are aggregated to each restriction fragment that represents potential DNA-DNA interacting sites (average length 413.0 bp). The extended restriction fragments with Hi-C reads are then filtered to include pairs with at least 2 reads supporting the interactions, for which one of the interacting pairs is a promoter (200 bp upstream from the TSS) and for which the non-promoter-interaction fragment overlaps with DNase-seq and known enhancer marks H3K4me1 and H3K27ac.

Given the high data biases associated with Hi-C assay, such as its relatively low resolution and experimental bias [61], this data could still be used with a tailored analysis pipeline to validate enhancer-gene predictions. Therefore, genome-wide Hi-C raw data applied to *Drosophila* embryonic nuclei, generated by [43], was downloaded and HIPPIE pipeline was applied.

HIPPIE outputs a Browser Extensible Data (BED) file for the interactions between gene promoters and Hi-C fragments, along with their corresponding *p*-values. These *p*-values represent the significance of read counts for each fragment-gene pair, calculated using the negative binomial distribution for the expected read counts for every fragment pair. HIPPIE reports a total of 1,924,222 interactions between 149,328 unique fragments and 9256 unique genes. When considering near-significant interactions only (*p*-value ≤ 0.1), the number of interactions drops to 239,933 between 98,376 unique fragments and 9240 unique genes. On average, a gene is assigned to 16 different fragments (10 when considering near-significant ones).

In order to calculate the overlap between our DHS-gene pairs and those reported by HIPPIE, its output was first filtered to contain only Hi-C fragments that overlap with all DHSs that do not overlap gene TSSs. A Hi-C fragment was considered to overlap a DHS only if at least 80% of the DHS is covered by the Hi-C fragment. Fragments interacting with gene promoters for only those genes that are in clusters used in this analysis were solely considered. After this, for each gene cluster, the number of DHS-gene pairs which overlap with Hi-C fragments and for which these fragments were linked to those same genes was counted, and represented by blue bars in Fig. 6 c. On average, ∼40% of DHS-gene pairs predictions are confirmed by Hi-C data, as shown by the blue line in Fig. 6 c. The BEDtools package GenomicRanges was used to find the overlap between BED coordinates [62].

To assess the significance of calculated overlap, random DHS-gene pairs were generated. For each gene in the analysis, six random DHSs, from DHSs within +/–50 kb around the gene TSS, were selected. The choice of six came from analyzing model prediction output where each gene is, on average, linked to six different DHSs. In addition, given that the predicted enhancer-gene pairs have skewed distribution towards shorter interactions, shown in Fig. 4 d, random DHS-gene pairs were sampled with controlled distance distributions, mimicking that of the predicted pairs. The same steps for calculating the overlap between predicted DHS-gene pairs and identified Hi-C fragment-gene pairs were applied on the random permuted data. This permutation was run 1000 times and the average overlap with Hi-C pairs was 15%, shown as the last blue bar in Fig. 6 c. This implies the significance of predictions for every gene cluster (*p*-value < 0.0001).

Similar procedures were then repeated, but considering only Hi-C fragment-gene pairs with significant *P* values (*p*-value ≤ 0.1). The average percentage overlap was ∼22%, with an average random overlap close to 4% (Additional file 1: Figure S5B).

### TF enrichment analysis in predicted regulatory regions

For further validation of predicted DHSs, we tested whether known motifs are enriched in the enhancer set selected for each cluster. We calculated enrichment scores for known position weight matrix (PWM) motifs in predicted DHSs for one cluster against DHSs predicted to regulate all other clusters, using AME [45]. We combined two highly curated motif datasets and used them as a database from which the AME algorithm selected enriched motifs. The first set represented binding specificities of 242 *Drosophila* TFs generated by applying the HT-SELEX protocol [46]. The other set included motifs from the OnTheFly database. Since the Mef2 motif, which is known to regulate muscle-related genes that we investigate in more detail, did not exist in any of the two databases, we added its PWM from [12].

To further narrow this down, we overlapped these results with important k-mers that significantly separated each cluster from others. After running the logistic regression classifier, important features that helped in classification were identified. We first calculated their z-scores based on the linear regression coefficient, then we ranked them accordingly. The first 20 features were used as input to TomTom [63] to search for motif matches against a set of databases. This resulted in different sets of known motifs, each representing DHSs predicted for specific clusters. Identified PWMs were analyzed and shown to match expression patterns of their associated clusters (the full table is shown in Additional file 4).

### Transgenic reporter assays

To validate enhancer-target predictions in vivo, two sets of candidates were chosen. The first set comprises published enhancers identified based on TF binding, but for which target genes were neither predicted nor investigated [12]. The second validation set comprises DHS regions likely to act as enhancers based on overlap with VT constructs [39]. The set 2 regions were PCR amplified from wild-type (yw) *D. melanogaster* using the primers listed below. The PCR products were *Eco*RI digested and non-directionally cloned into the P-element reporter vector pH-Stinger [64] and verified by diagnostic digest and Sanger sequencing. All constructs were integrated via P-element transgenesis according to standard protocols [65]. Between two and five independent transgenic lines were obtained and evaluated for each construct to control for position effects. Embryos of mixed developmental stages were collected from transgenic lines, formaldehyde cross-linked, and tested by whole-mount in situ hybridization (ISH) using antisense RNA probes against the reporter genes (lacZ for set 1, or GFP for set 2) and predicted target genes. RNA probes labeled with digoxigenin-, biotin-, or fluorescein-uridine triphosphates (UTPs) for co-visualization according to standard protocols, similar to [66], were generated. Visualization was performed fluorescently via tyramide signal amplification (TSA kit, Perkin Elmer) after labeling of the individual haptens with primary antibodies (Anti-Fluorescein-POD, Anti-Digoxigenin-POD, or Anti-Biotin-POD, Roche) as appropriate. Co-visualization allows for the direct in situ comparison of the spatio-temporal expression patterns driven by the DHS candidates identified in relation to the expression domain of predicted target genes. Images were acquired in Z-stacks on either a Leica DMi8 wide field or a Zeiss LSM 700 confocal scanning system; wide field images were software deconvolved.

Primers for amplification of set 2:

- candidate 1
  - DHS chr2L_8851915_stage_9 ; location = [668bp=2L:8851915-8852583] cloned: chr2L:8851854-8852589
  - Fwd = #544 : nnngaattcCCAATCAAAATAAATGGCTAC
  - Rev = #545 : nnnngaattcGCGATGTCAAAGGTCTTAAC
- candidate 2
  - DHS chr3L_14146268_stage_9 ; location = [238bp=3L:14146268-14146506] cloned chr3L:14146267-14146516
  - Fwd = #546 : nnnngaattcTGTCTAACTGTGCATCCCTG
  - Rev = #547 : nnnngaattcACAATCCCGGATACAAAAGG
  Note that candidate 2 has not been tested, because it failed to be identified as a candidate after the prediction model was refined
- candidate 3
  - DHS chr3L_14146763_stage_5 ; location = [398bp=3L:14146763-14147161] cloned: chr3L:14146754-14147167
  - Fwd = #548 : nnnngaattcGCTAATCGTTCGCCTTCTCG
  - Rev = #549 : nnnngaattcCCTTGCAGGTCAGATGTCC
- candidate 4
  - DHS chr3L_3976412_stage_9 ; location = [691bp=3L:3976412-3977103] cloned chr3L:3976411-3977103
  - Fwd = #550 : nnnngaattcCTACGTGGATGAGCTCC
  - Rev = #551 : nnnngaattcAGCTAAGAGGATGTCATGAAC
- candidate 5
  - DHS chr3L_3978213_stage_9 ; location = [392bp=3L:3978213-3978605] cloned chr3L:3978207-3978615
  - Fwd = #552 : nnnngaattcGAGTGCATCGCTTTTACC
  - Rev = #553 : nnnngaattcCGAATCGTGTGTTGAGATAG

## Additional files

Additional file 1 Supplementary figures. (PDF 9185 kb)

Additional file 2 Materials and methods supplement. This text explains McEnhancer in more details; phase I and II of McEnhancer, model selection parameters and cross validation. (PDF 236 kb)

Additional file 3 McEnhancer predicted DHSs for each cluster. This table shows the predicted DHSs for each cluster, along with its associated gene. Columns represent DHS coordinates (chr, start, end), DHS ID linked to its associated gene, and number of times each DHS was selected in the model’s pairwise comparisons against other clusters. (TXT 778 kb)

Additional file 4 TF motifs enriched in predicted DHSs. This table shows enriched PWMs in predicted DHSs for each cluster using the AME motif enrichment algorithm. (XLSX 28 kb)

Additional file 5 Table showing McEnhancer validation of long-range interactions reported in REDfly. This table shows all long-range interactions reported in REDfly that overlap our DHSs, selecting only those that were assigned to genes in included in our analysis, but these genes are not the closest. Columns of this table are: CRMs coordinates from Redfly, their assigned genes, whether such gene is has unique or multiple expression pattern, closest genes, coordinated of McEnhancer predicted DHSs that overlap each CRM, and whether McEnhancer prediction is correct, and a comment in case the assigned genes are different. (XLSX 59 kb)

## Abbreviations

AuROC: Area under receiver operating characteristic curve
CRM: Cis-regulatory module
DHS: DNaseI hypersensitive site
EM: Expectation-maximization
HMM: Hidden Markov model
IMM: Interpolated Markov chain model
REDfly: Regulatory Element Database for Drosophila
TF: Transcription factor
TFBS: Transcription factor binding site
TSS: Transcription Start Site
VT: Vienna Tiles
