# Supplementary material for: McEnhancer: predicting gene expression via semi-supervised assignment of enhancers to target genes
Source: Genome Biol. 2017 Oct 26;18:199. doi: 10.1186/s13059-017-1316-x (PMC5657048; doi:10.1186/s13059-017-1316-x)
Supplement: Supplementary file 1 — Supplementary figures. (PDF 9185 kb) [file 13059_2017_1316_MOESM1_ESM.pdf]

Figure S1: Pseudocode of McEnhancer

input : sequences of known DHSs regulating genes belonging to specific expression clusters
sequences of all DHSs in a window of +/-50 kb around TSSs of each gene in cluster c.
output : DHSs regulating genes in cluster c are assigned a *yes* label.

begin
  for $i \in clusters$ **do**
    initialize: set D_i^{lc} = known DHSs for cluster c
      set D_i^{l} = known DHSs for cluster i (negative/null model)
      set D_i^{uc} = all other DHSs in +/-50 kb window around TSSs for genes in cluster c.
    // **E-Step**
    $positiveIMM$ = train a 3-order IMM from D_i^{lc} ;
    $negativeIMM_i$ = train a 3-order IMM on known DHSs for cluster i (D_i^l set);
    repeat
      for $(DHS^u \in D_i^{uc})$ **do**
        calculate loglikelihood DHS^u_{pos} from positive-IMM;
        calculate loglikelihood DHS^u_{neg} from negative-IMM;
        if $(DHS^u_{pos} > DHS^u_{neg})$ **and**
          $(DHS^u_{pos} - DHS^u_{neg} > rejectClassThreshold)$ **:**
          add DHS to D_i^{lc} ;
          remove DHS from D_i^{uc} ;
      end
      // **M-Step**
      $positiveIMM$ = update positive-IMM parameters based on updated D_i^{lc} set ;
      for $(DHS^l \in D_i^{lc})$ **do**
        calculate loglikelihood DHS^l_{pos} from positive-IMM;
        calculate loglikelihood DHS^l_{neg} from negative-IMM;
        if $(DHS^l_{pos} > DHS^l_{neg})$ **:**
          keep DHS in D_i^{lc} ;
        else:
          remove DHS from D_i^{lc} ;
          add DHS to D_i^{uc} ;
      end
    until D_i^{uc} does not change **or** maximum number of iterations exceeded;
  end
  `getDHSsSelectedAtLeast10Times`(D^{lc})
end

Figure S2: Number of labeled DHS-gene pairs. No. of known distal DHS-gene pairs overlapping REDfly and VT, which are used for model initialization.

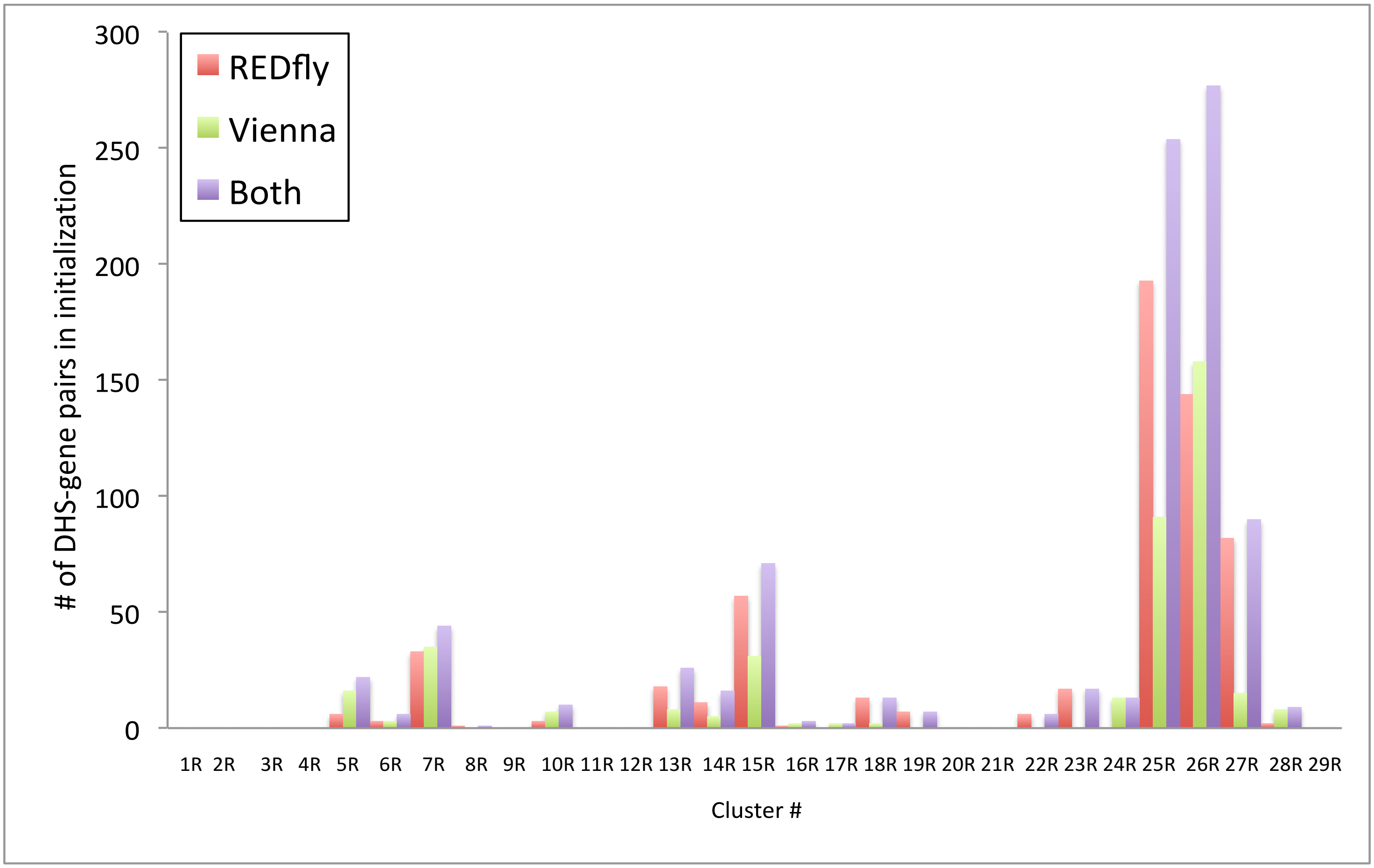

Figure S3: No. of genes that are within 100kb of each other per cluster

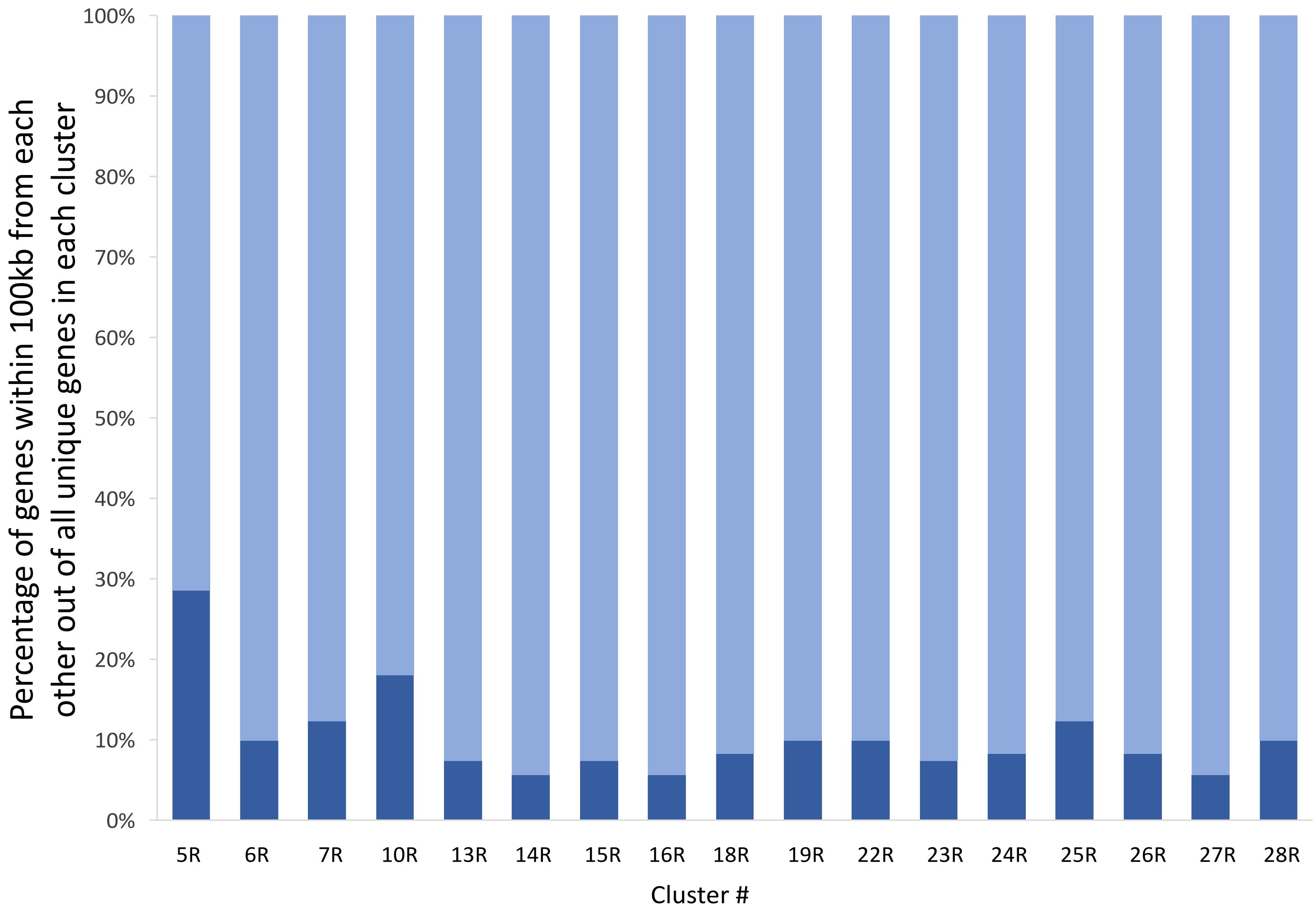

Figure S4: Computational validation of McEnhancer predictions by initialization with REDfly exclusively. (A) Overlap of selected DHSs when initializing McEnhancer by REDfly only, with the full set of VT DHSs assigned to each cluster. (B) Distribution of enhancer-gene distances in the VT set, and of the recovered subset when initializing on REDfly only.

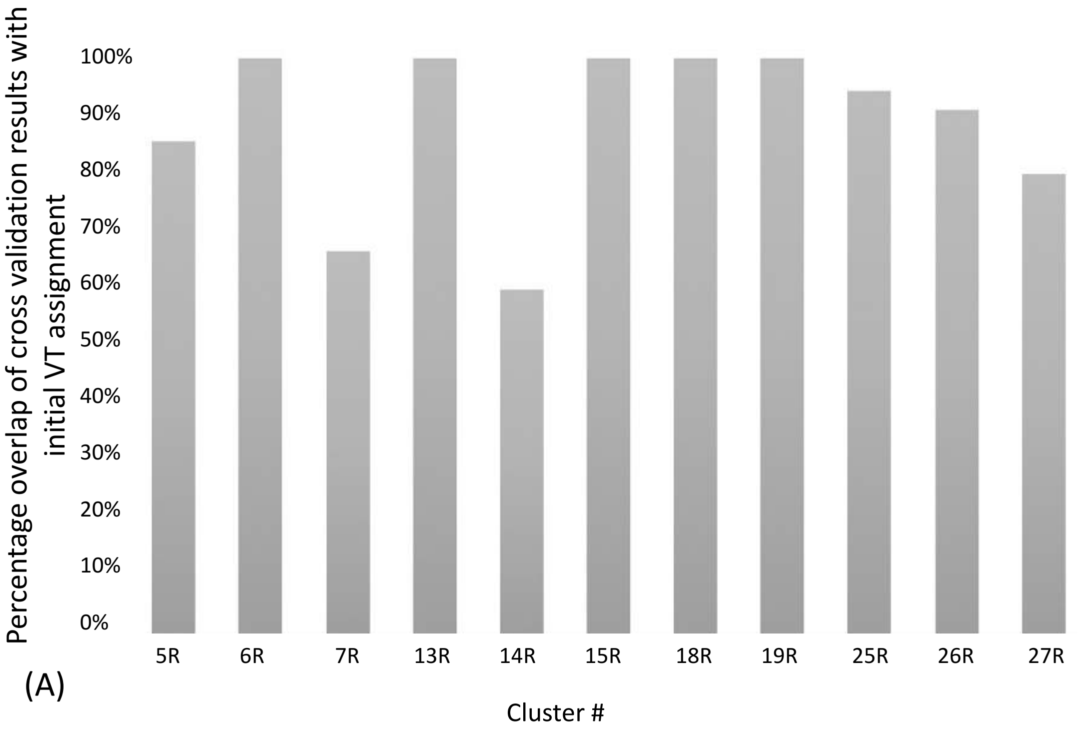

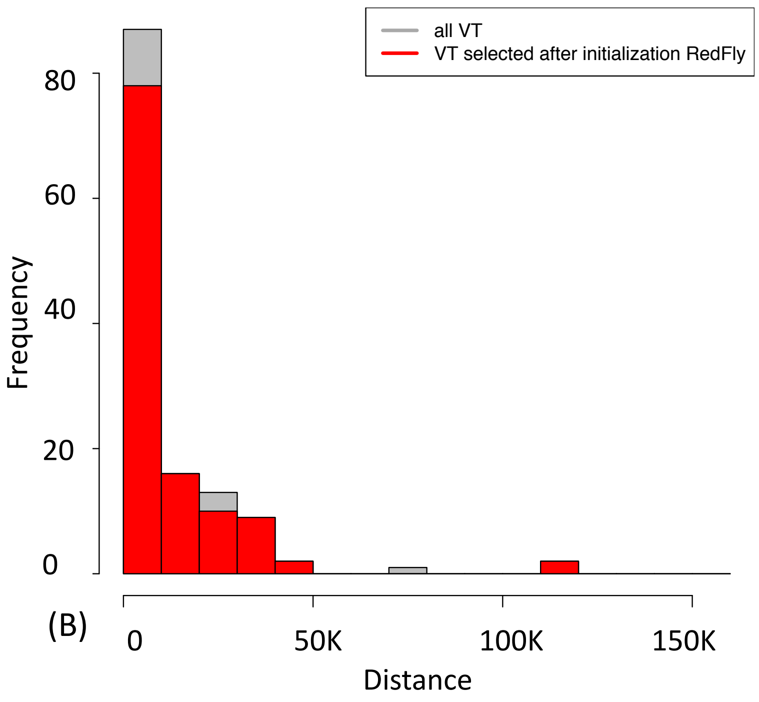

Figure S5: Average DHS accessibility for McEnhancer predictions and overlap with significant Hi-C fragments. (A) The average DHS accessibility for the McEnhancer selected DHSs and other control groups. DHS peak signal was averaged across each of the four groups and centered over DHS midpoint. (B) Validation of predicted DHS-gene pairs by overlapping with significantly identified fragment-promoter Hi-C interactions. We used the default setting of HIPPIE for setting significance threshold for interaction to be P-value < 0.0001. Percentages represented by this overlap with respect to filtered predicted DHS-gene pairs are shown by the dotted line.

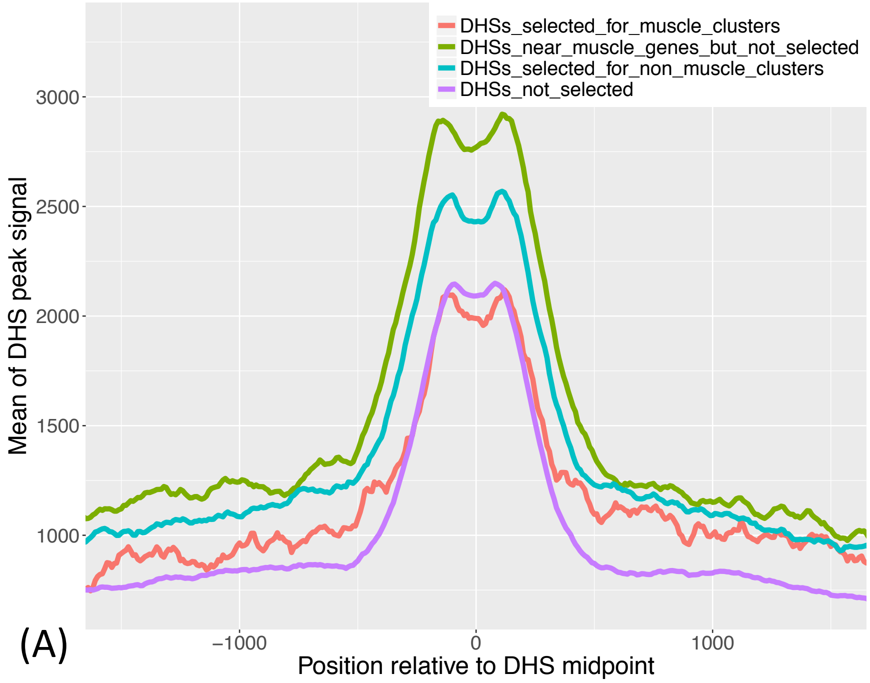

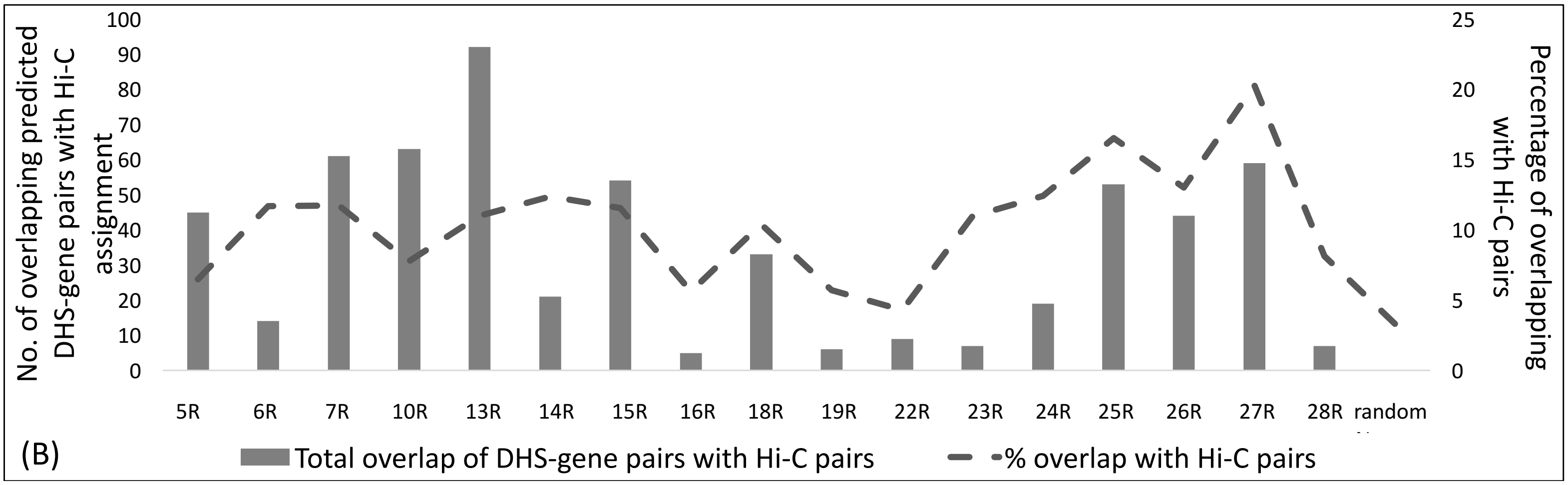

Figure S6: TFs Heatmap showing enriched TFs in each gene cluster. Using AME motif enrichment algorithm, enriched TFs based on reported binding preferences were identified, and their corresponding Pvalue score are shown on the heatmap (upper panel). Overlapping 5-mers and their matched TFs are shown on the bottom panel, with a black squares means "match" and a white one "no match".

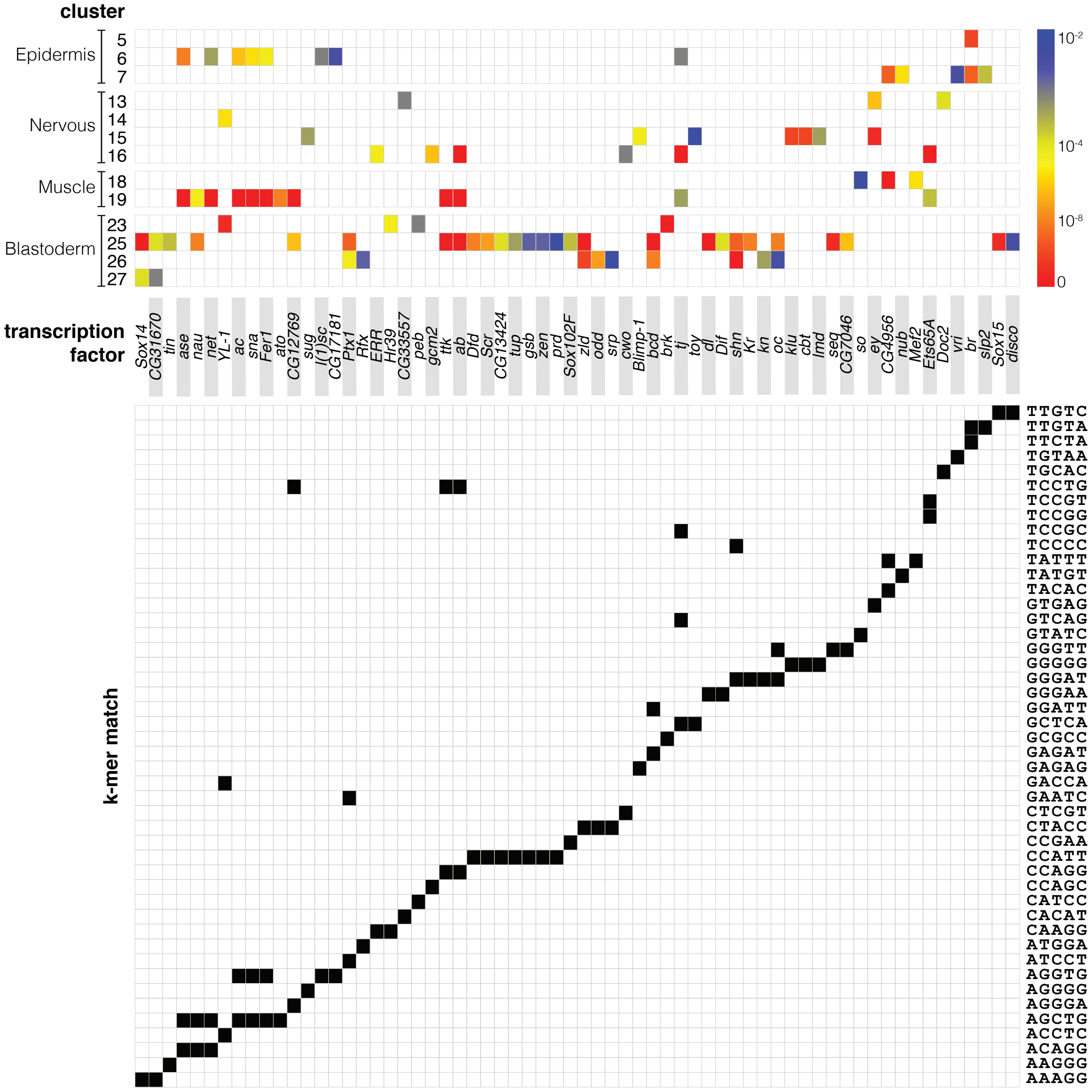

Figure S7: Experimental validation for selected candidates. Figure structured as Figure 8. Genomic and in-vivo reporter data is shown for CRM6053 (A), CRM5481 (B), CRM3775 (C), CRM4515 (D), and candidate 3 (E). Embryo developmental stages are indicated, orientation is anterior left. All embryos are lateral views ventral down, except B'-st.15, which is a dorsal view and D'-st.9, which is a ventral view.

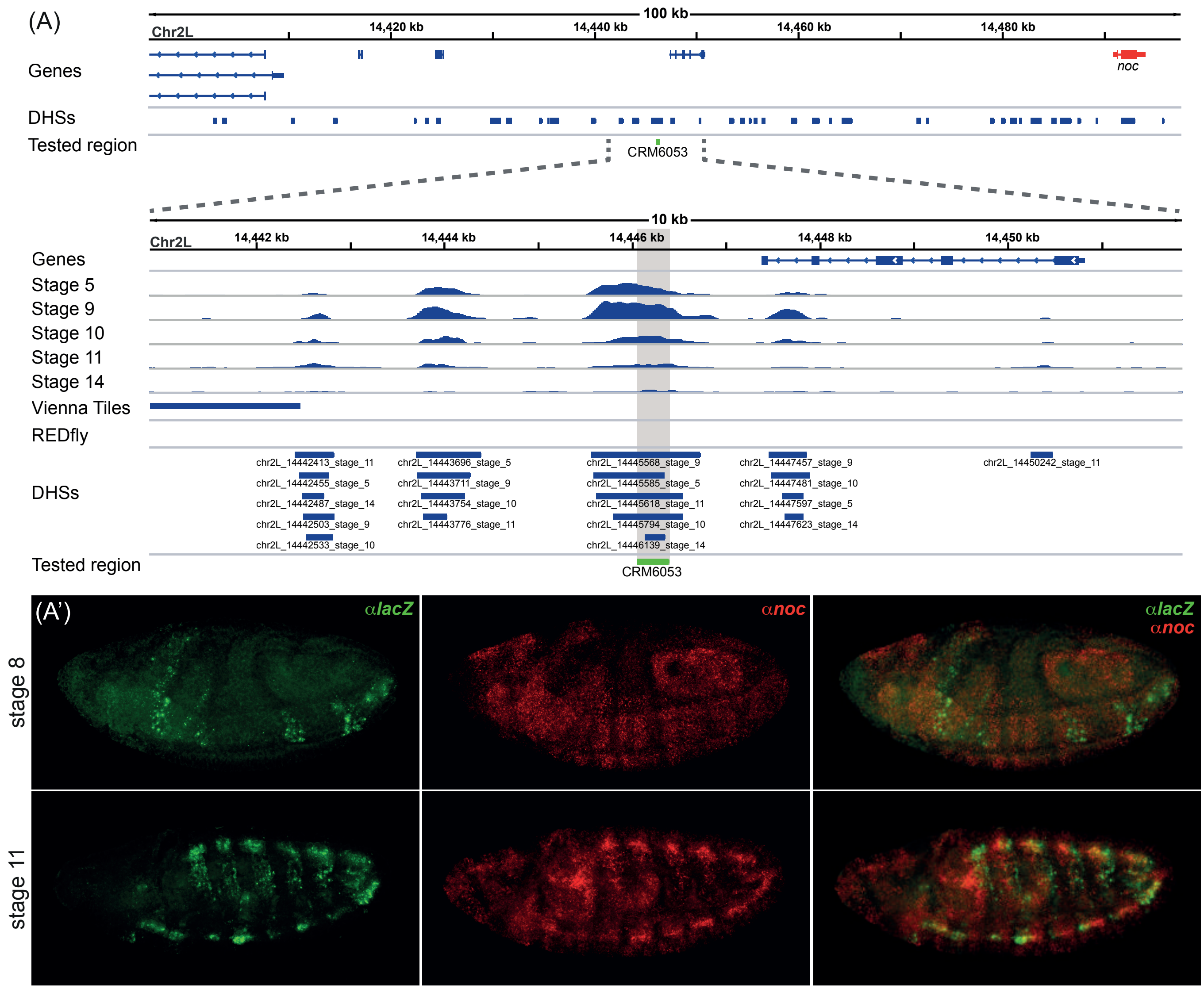

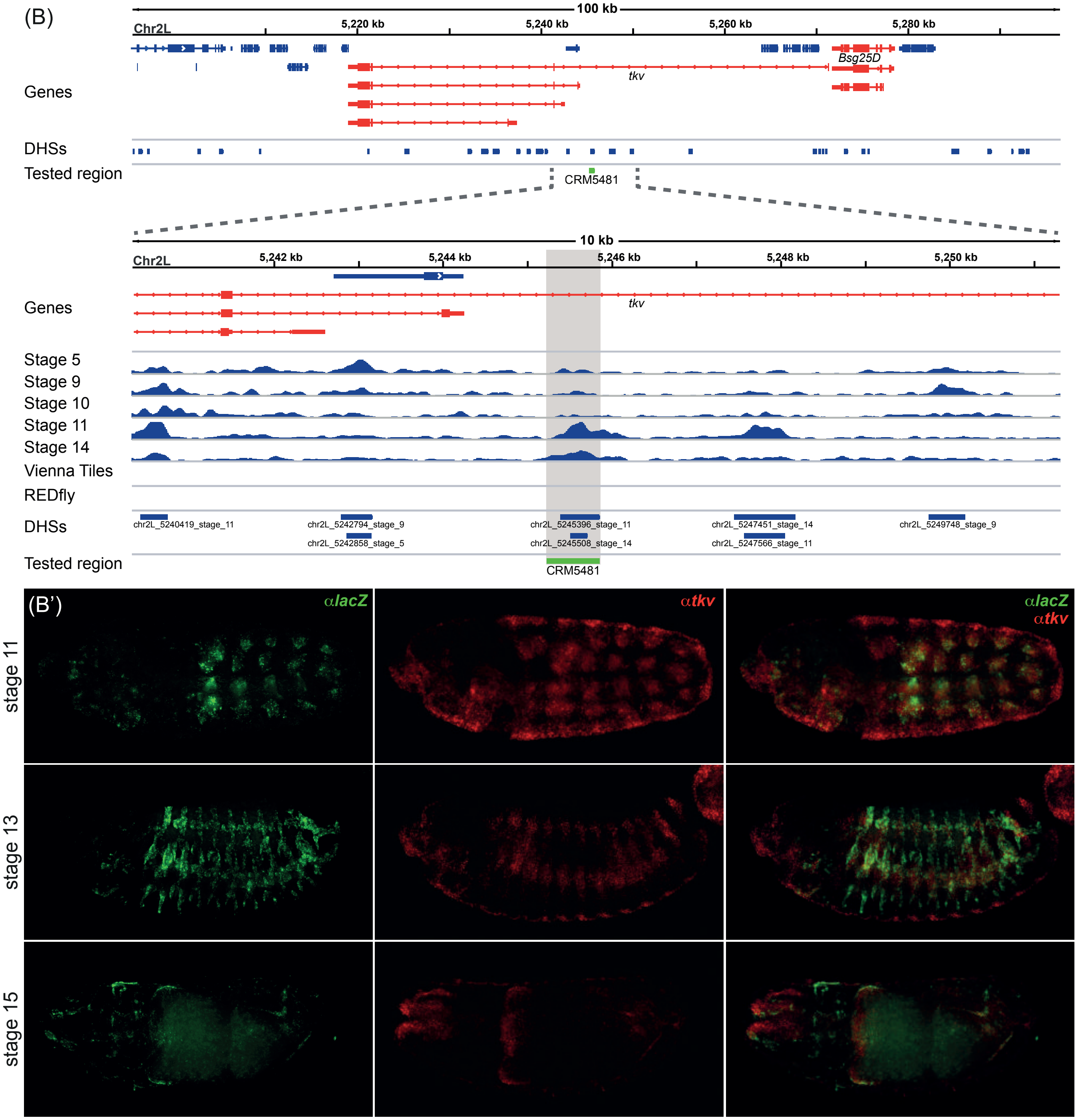
(B)
100 kb
Chr2L
5,220 kb
5,240 kb
5,260 kb
5,280 kb
Genes
Bsg25D
tkv
DHSs
Tested region
CRM5481
10 kb
Chr2L
5,242 kb
5,244 kb
5,246 kb
5,248 kb
5,250 kb
Genes
tkv
Stage 5
Stage 9
Stage 10
Stage 11
Stage 14
Vienna Tiles
REDfly
DHSs
chr2L_5240419_stage_11
chr2L_5242794_stage_9
chr2L_5242858_stage_5
chr2L_5245396_stage_11
chr2L_5245508_stage_14
chr2L_5247451_stage_14
chr2L_5247566_stage_11
chr2L_5249748_stage_9
Tested region
CRM5481
(B')
αlacZ
αtkv
αlacZ
αtkv
stage 11
stage 13
stage 15

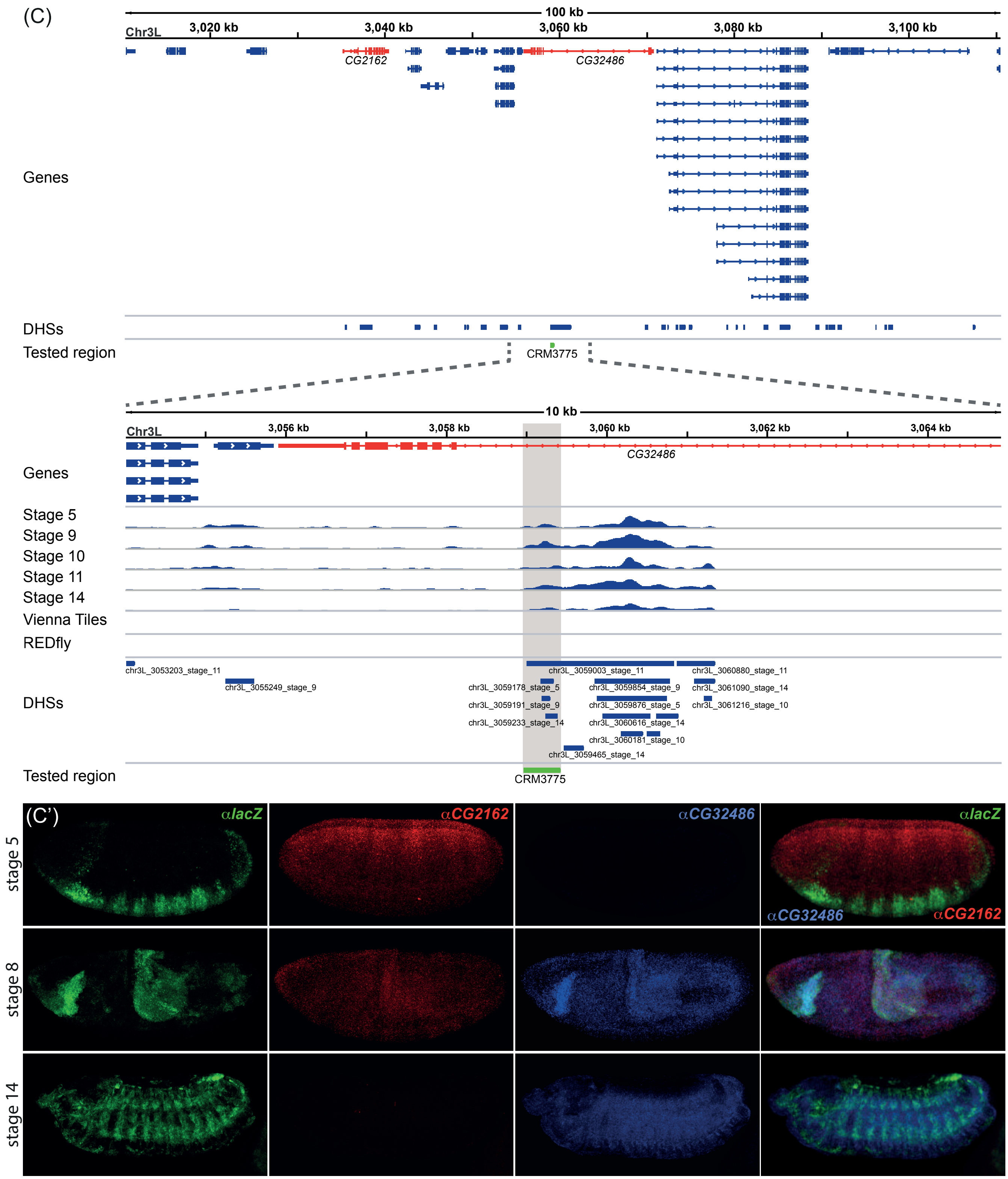
(C)
100 kb
Chr3L
3,020 kb
3,040 kb
3,060 kb
3,080 kb
3,100 kb
CG2162
CG32486
Genes
DHSs
Tested region
CRM3775
10 kb
Chr3L
3,056 kb
3,058 kb
3,060 kb
3,062 kb
3,064 kb
CG32486
Genes
Stage 5
Stage 9
Stage 10
Stage 11
Stage 14
Vienna Tiles
REDfly
DHSs
chr3L_3053203_stage_11
chr3L_3055249_stage_9
chr3L_3059178_stage_5
chr3L_3059191_stage_9
chr3L_3059233_stage_14
chr3L_3059003_stage_11
chr3L_3059854_stage_9
chr3L_3059876_stage_5
chr3L_3060616_stage_14
chr3L_3060181_stage_10
chr3L_3059465_stage_14
chr3L_3060880_stage_11
chr3L_3061090_stage_14
chr3L_3061216_stage_10
Tested region
CRM3775
(C')
αlacZ
αCG2162
αCG32486
αlacZ
αCG32486
αCG2162
stage 5
stage 8
stage 14

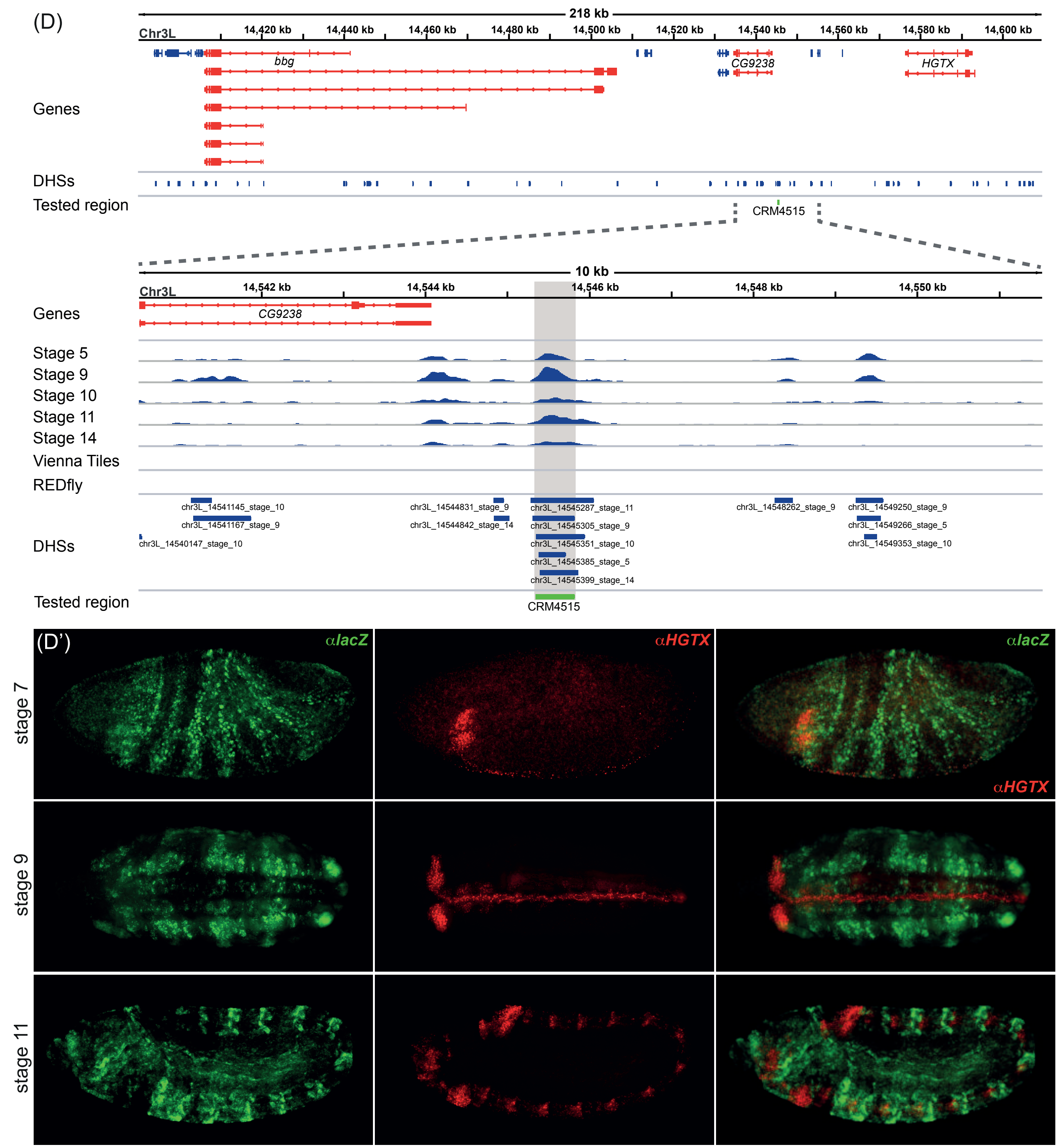
(D)
218 kb
Chr3L
14,420 kb
14,440 kb
14,460 kb
14,480 kb
14,500 kb
14,520 kb
14,540 kb
14,560 kb
14,580 kb
14,600 kb
Genes
bbg
CG9238
HGTX
DHSs
Tested region
CRM4515
10 kb
Chr3L
14,542 kb
14,544 kb
14,546 kb
14,548 kb
14,550 kb
Genes
CG9238
Stage 5
Stage 9
Stage 10
Stage 11
Stage 14
Vienna Tiles
REDfly
DHSs
chr3L_14541145_stage_10
chr3L_14541167_stage_9
chr3L_14540147_stage_10
chr3L_14544831_stage_9
chr3L_14544842_stage_14
chr3L_14545287_stage_11
chr3L_14545305_stage_9
chr3L_14545351_stage_10
chr3L_14545385_stage_5
chr3L_14545399_stage_14
chr3L_14548262_stage_9
chr3L_14549250_stage_9
chr3L_14549266_stage_5
chr3L_14549353_stage_10
Tested region
CRM4515
(D')
αlacZ
αHGTX
αlacZ
αHGTX
stage 7
stage 9
stage 11

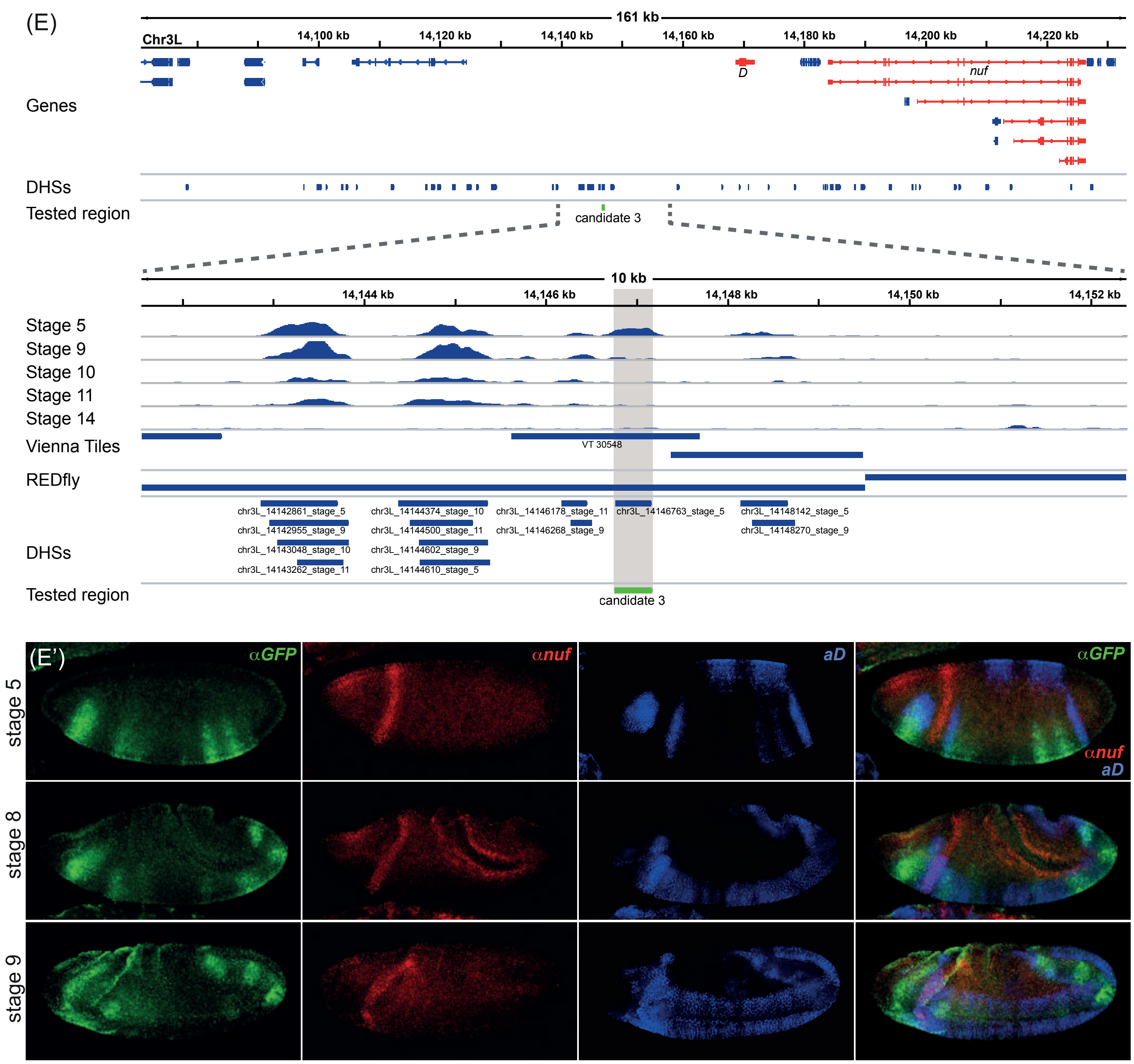

(E)
161 kb
Chr3L
14,100 kb
14,120 kb
14,140 kb
14,160 kb
14,180 kb
14,200 kb
14,220 kb
D
nuf
Genes
DHSs
Tested region
candidate 3
10 kb
14,144 kb
14,146 kb
14,148 kb
14,150 kb
14,152 kb
Stage 5
Stage 9
Stage 10
Stage 11
Stage 14
Vienna Tiles
VT 30548
REDfly
chr3L_14142861_stage_5
chr3L_14142955_stage_9
chr3L_14143048_stage_10
chr3L_14143262_stage_11
chr3L_14144374_stage_10
chr3L_14144500_stage_11
chr3L_14144602_stage_9
chr3L_14144610_stage_5
chr3L_14146178_stage_11
chr3L_14146268_stage_9
chr3L_14146763_stage_5
chr3L_14148142_stage_5
chr3L_14148270_stage_9
DHSs
Tested region
candidate 3
(E')
αGFP
αnuf
aD
αGFP
αnuf
aD
stage 5
stage 8
stage 9
