# Supplementary material for: McEnhancer: predicting gene expression via semi-supervised assignment of enhancers to target genes
Source: Genome Biol. 2017 Oct 26;18:199. doi: 10.1186/s13059-017-1316-x (PMC5657048; doi:10.1186/s13059-017-1316-x)
Supplement: Supplementary file 2 — Materials and methods supplement. This text explains McEnhancer in more details; phase I and II of McEnhancer, model selection parameters and cross validation. (PDF 236 kb) [file 13059_2017_1316_MOESM2_ESM.pdf]

Additional file 2

Semi-supervised interpolated Markov chain model

The goal of this step is to assign a class label for each DHS indicating the gene expression cluster(s) that this DHS is associated with. After initialization, for each group of genes belonging to the same expression cluster, sequences of their associated known DHSs are assumed to represent regulatory sequences required for regulation of this specific expression pattern. A 3-order IMM model is trained on sequences of this initial assignment, positive model. Another IMM model is trained on a null model. This null model represents DHS sequences that are known to regulate one other gene expression cluster.

When presented with a sequence for an unlabeled DHS, a DHS that does not have a class label assigned to, the model evaluates the likelihood of this sequence being generated by the trained positive model and contrast it to the likelihood of being generated by the null model. This DHS is considered to be regulating the positive expression cluster (assigned to labeled DHSs) if its positive computed likelihood is greater than its negative/null likelihood.

This is a semi-supervised learning problem, in which a small set of labeled examples is known, but a large set of all other DHSs have no class labels (unlabeled examples). McEnhancer assigns a label to each of the unlabeled examples in an iterative manner. It first learns the sequences of the enriched motifs (k-mers) from the few known DHSs for a group of genes having similar expression patterns. This is achieved by building two 3-order interpolated Markov (IMM) models from the initially assigned DHSs that overlap REDfly and VT; one for a given gene cluster (positive model) and one for the other cluster against which the first cluster is compared (negative model). A schematic representation of the model is shown in given in Methods

In order to guarantee convergence, an expectation-maximization (EM) algorithm is then applied to predict other DHSs with similar sequence composition in a semi-supervised learning technique. The EM algorithm is a general algorithm for maximum likelihood estimation (MLE) with missing data. It is used to infer the label for each DHS. In each iteration, McEnhancer updates the IMM positive model to incorporate the newly assigned labeled examples, and remove DHSs that changed their label. Pseudocode for the model is displayed in Suplementary Figure 2.

In an attempt to protect a given gene cluster from labeled DHSs with slightly higher likelihood for the positive example, but not sufficiently high to be specific to such cluster, McEnhancer allows for a “reject option”. This is equivalent to “I don’t know” which abstains hazardous decisions [1]. This is better than assigning a positive label for a DHSs with low sequence content and generating much noise that would interfere with further assignments.

For each gene cluster, this model runs 16 times, each time against a different gene cluster, to allow the algorithm to pick DHSs with sequences specific to the given positive gene cluster. SRILM toolkit was used for IMM implementation [2]. Since the number of known examples used in training is very small, it is

advised to use Witten-Bell discounting/smoothing. As a final step, selected DHSs from the 16 pairwise runs against each of the other clusters are grouped together. Only DHSs that gets selected at least 60% of the times (10 out of the 16 pairwise runs) are assigned to this expression cluster.

Sparse logistic regression classifier

To determine how well selected DHSs are specific for a given gene cluster, sparse logistic regression classifiers are used. These classifiers balance the use of many available features against model complexity, ending up with a selection of a small subset of features that are used in the classification. Sparse logistic regression classifiers minimizes an objective function that is a linear combination of the sum of squared residuals and the $\ell 1$ norm of the weights [3]. Since the goal is to predict expression pattern of each gene expression clusters given the assigned DHSs, this classifier will be used to measure the power of assignment of DHSs to their gene clusters.

Given two groups of DHS sequences that are predicted to regulate two different gene groups, the classifier performs 4-fold cross validation, with data shuffled before each iteration. In each round, three parts of the data are further divided into six parts, one of which is used as the validation set to learn the hyperparameter. The classifier uses the score for all 5-mers, by counting the frequencies of occurrences of each of the 5-mers in the input DHS sequences for both positive and negative examples. Each of the 5-mers is used as a separate feature. A k-mer and its reverse complement are treated as a single feature with counts representing the frequencies of occurrences of the k-mer and its reverse complement. A total of 10 iterations are conducted and the average area under the receiver operating characteristics (AuROC) for classification is computed. AuROC is a measure of classification performance, where a value of 0.5 indicates random assignments and 1.0 indicates perfect classification. Overlapping DHSs assigned to the same gene are merged into a single DHS, so as to avoid double counting of kmer features. In case of having unbalanced datasets, where number of positive examples is greater than negative examples or vise versa, the classifier randomly samples from the class with larger number of examples the same number of examples as that of the smaller class.

As a general note, since most of genes are generally regulated by more than one DHS, there exists two normalization alternatives. The first is to assume that each DHS regulates the gene separately, therefore k-mer frequencies are normalized by the length of each DHS. The second alternative is to assume that DHSs associated with a gene play a collaborative role and thus normalize k-mer frequencies by the total length of DHSs. Both alternatives generally gave similar performance in classification. Results reported in this work adopt the second view.

Model assumptions

Model assumptions include:

- DHSs overlapping promoter regions often have different properties than those overlapping distal regions. McEnhancer accounts for different effects of the two regulatory processes. It is already accepted that most of regulatory elements located in a core promoter region of a gene influence expression of their strictly downstream gene. Therefore, DHSs overlapping promoter regions are assigned to genes whose TSSs they overlap. The model then focuses on assigning distal DHSs to their target genes. All classifiers that are built to predict specific expression patterns use features from distal DHSs only.
- In *Drosophila melanogaster*, the majority of known enhancers are within +/-20 kb of their target genes, while a few have been detected to be around 35kb from their target genes [4]. Therefore, search space of the model is restricted to include DHSs found in +/-50kb around TSSs of each of the corresponding genes.

Phase I and phase II for McEnhancer

Genes with restricted expression patterns were grouped into 29 different clusters, with 59% belonging to exactly one expression cluster (unique genes), while 31% share multiple clusters (common genes). Out of these 29 clusters, only 17 clusters were considered since their genes have associated known distal DHSs valid for initialization. Since genes with multiple expression patterns could be regulated by different enhancers, and due to the limited number of known labeled Distal-DHSs, McEnhancer was run on each of these clusters in two phases. Phase I predicts Distal-DHSs regulating unique genes only, while phase II predicts distal regulating DHSs for common genes. In phase II, the model uses predicted Distal-DHSs from phase I to initialize its parameters. This separation allowed the model to learn best sequences specific for the gene cluster in consideration, without being distracted by sequences important for other expression patterns but not the one considered (due to the gene belonging to more than one cluster).

These two phases were run in pairwise comparisons. Each expression cluster was modeled against each of the 16 other clusters. Then selected Distal-DHSs were grouped together, and Distal-DHSs that got selected in at least 10 models for a given cluster were only considered. Different cutoffs (DHSs had to be selected in 10 or more different models) were tested, and numbers of DHSs that were selected at different cutoffs were documented per each cluster, and their frequencies displayed in Additional file 2. Grouping all clusters together resulted in a histogram with the shape in Figure 1A. This histogram shows almost a bimodal distribution with two peaks, at 5 & 10. The first distribution represents Distal-DHSs that were selected in few comparisons. These Distal-DHSs might still be necessary for regulating the given expression cluster but they are not uniquely specific to that expression pattern when compared against other expression clusters. The second distribution represents DHSs that are uniquely and significantly specific to this given cluster. Therefore, the value

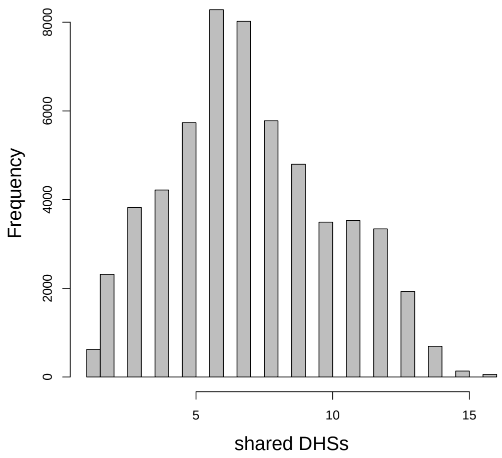

(A)

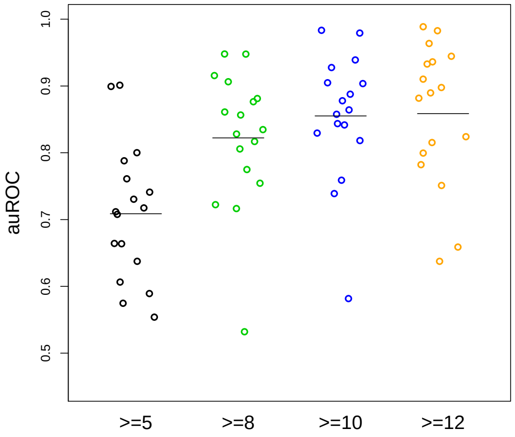

(B)

Figure 1
Grouped common DHSs frequencies with classification performance for different overlap cutoffs.
(A) Histograms showing number of Distal-DHSs selected at each cutoff for all clusters grouped together. (B) Sparse logistic regression classification showing different overlap cutoffs for selected Distal-DHSs per cluster classified against ubiquitous.

10 was chosen as a threshold, and Distal-DHSs selected at least 10 times were considered.

Furthermore, Distal-DHSs selected at different cutoffs, at least 5, 8, 10 and 12 times were classified against known Distal-DHSs regulating ubiquitously expressed genes. For each cutoff, AuROC for the average classification output per cluster displayed in Figure 1B. Distal-DHSs selected at least 10 times gave an average AuROC 86%.

Model parameter selection for McEnhancer

Cross validation (CV) is usually used to avoid overfitting. In order to decide on the maximum IMM order, CV was applied on the set of DHSs used in initialization. For each cluster, DHSs were divided into four parts to perform 4-fold cross validation; three parts were used for training and one part for testing. Interpolated Markov orders (1, 2, . . . , 6) were tested and the overall log likelihood values for each order per class are documented in Table 1.

A total of 10 iterations for each 4-fold cross validation were performed, with

Table 1
Model selection parameter for for interpolated Markov chain order.

MC order cluster	1	2	3	4	5
5R	-51.288	-51.0360	-50.958	-50.956	-50.964
6R	-29.197	-29.2840	-29.449	-29.491	-29.492
7R	-88.714	-87.9620	-87.567	-87.512	-87.511
10R	-51.846	-51.5903	-51.537	-51.542	-51.549
13R	-54.618	-54.0810	-53.835	-53.807	-53.819
14R	-73.015	-72.485	-72.324	-72.318	-72.319
15R	-56.247	-55.948	-55.817	-55.804	-55.815
16R	-78.802	-79.521	-80.316	-80.472	-80.382
18R	-64.123	-63.765	-63.660	-63.655	-63.648
19R	-33.789	-33.772	-33.842	-33.862	-33.849
22R	-32.047	-31.983	-32.040	-32.061	-32.055
23R	-51.507	-51.365	-51.373	-51.387	-51.395
24R	-66.021	-65.862	-65.856	-65.867	-65.868
25R	-73.132	-72.870	-72.751	-72.738	-72.751
26R	-69.885	-69.668	-69.572	-69.562	-69.574
27R	-70.422	-70.099	-69.953	-69.938	-69.951
28R	-59.492	-58.875	-58.642	-58.623	-58.624
average	-55.992	-55.713	-55.620	-55.567	-55.506

the data shuffled before each iteration. As expected, the number of initial DHSs affected the likelihood per each. To be able to compare them, log likelihood was normalized by dividing by the number of DHSs involved. The average log likelihood probability for each IMM order is shown in Figure 2A. Log likelihood values reach a plateau at order 3, after which it flattens. Yet, it is interesting to see the effect of interpolation in avoiding overfitting, by not making the curve drastically decrease afterwards. Based on that, IMM of order 3 will be used to build the model. As it was shown in Supplementary Figure 4, for most classes, the number of DHSs used in initialization is pretty minimal, therefore, reaching 3-order MC without overfitting is encouraging.

Regarding the best mixture weights for each order, again 4-fold CV was applied on different combinations for (λ_3, λ_2, λ_1, λ_0), one for each order/model. The different combinations and average log likelihood values among all gene clusters are shown in Table 2 and Figure 2B. Since differences in average log likelihood values are not that huge, some combinations were compared and the one that gave the highest value was chosen, where $(\lambda_3, \lambda_2, \lambda_1, \lambda_0) = (0.3, 0.25, 0.25, 0.2)$.

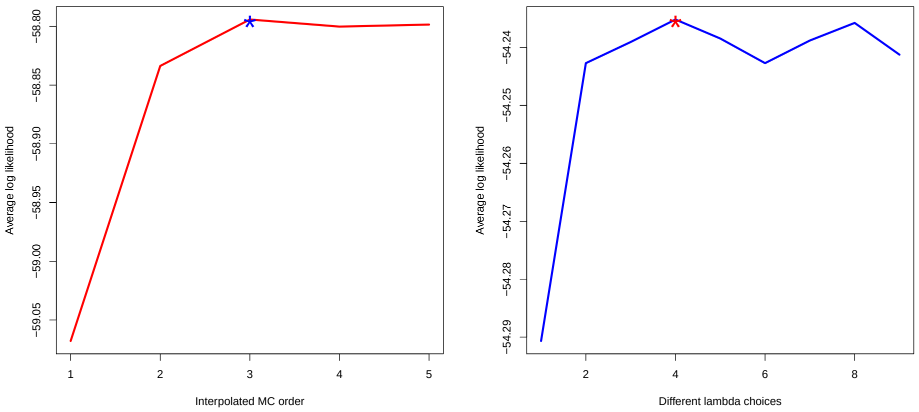

(A) **(B)**

Figure 2
Parameters for model selection.
Cross validation output for selecting model parameters: (A) IMM order, (B) lambda.

Table 2
Model selection parameters for interpolated Markov model weights (λs).

(λ_3, λ_2, λ_1, λ_0)<br>avg. log likelihood	(0.3, 0.1, 0.2, 0.4)<br>-54.290	(0.25, 0.25, 0.25, 0.25)<br>-54.242
(0.2, 0.3, 0.4, 0.1)<br>-54.239	**(0.3, 0.25, 0.25, 0.2)**<br>**-54.235**	(0.25, 0.25, 0.3, 0.2)<br>-54.238
(0.2, 0.3, 0.25, 0.25)<br>-54.2427	(0.35 ,0.3, 0.2, 0.15)<br>-54.238	(0.4, 0.15, 0.35, 0.1)<br>-54.236
(0.4, 0.2, 0.35, 0.05)<br>-54.241		

References

[1] Hanczar, B., Sebag, M.: Combination of one-class support vector machines for classification with reject option. In: Machine Learning and Knowledge Discovery in Databases, pp. 547–562 (2014)

[2] Stolcke, A., *et al.*: Srilm-an extensible language modeling toolkit. In: INTERSPEECH (2002)

[3] Kim, S.-J., Koh, K., Lustig, M., Boyd, S., Gorinevsky, D.: An interior-point method for large-scale l 1-regularized least squares. Selected Topics in Signal Processing, IEEE Journal of **1**(4), 606–617 (2007)

[4] Cléard, F., Moshkin, Y., Karch, F., Maeda, R.K.: Probing long-distance regulatory interactions in the drosophila melanogaster bithorax complex using dam identification. Nature genetics **38**(8), 931–935 (2006)
